# Fluorescence assay for simultaneous quantification of CFTR ion-channel function and plasma membrane proximity

**DOI:** 10.1074/jbc.RA120.014061

**Published:** 2021-01-13

**Authors:** Stella Prins, Emily Langron, Cato Hastings, Emily J. Hill, Andra C. Stefan, Lewis D. Griffin, Paola Vergani

**Affiliations:** 1 Department of Neuroscience, Physiology, and Pharmacology, University College London, London, United Kingdom; 2 CoMPLEX, University College London, London, United Kingdom; 3 Natural Sciences, University College London, London, United Kingdom

**Keywords:** gating, anion transport, conductance, cystic fibrosis, fluorescence, microscopic imaging, intracellular trafficking, protein stability, molecular pharmacology, VX-770, cystic fibrosis transmembrane conductance regulator (CFTR), ABC transporter

## Abstract

The cystic fibrosis transmembrane conductance regulator (CFTR) is a plasma membrane anion channel that plays a key role in controlling transepithelial fluid movement. Excessive activation results in intestinal fluid loss during secretory diarrheas, whereas *CFTR* mutations underlie cystic fibrosis (CF). Anion permeability depends both on how well CFTR channels work (permeation/gating) and on how many are present at the membrane. Recently, treatments with two drug classes targeting CFTR—one boosting ion-channel function (potentiators) and the other increasing plasma membrane density (correctors)—have provided significant health benefits to CF patients. Here, we present an image-based fluorescence assay that can rapidly and simultaneously estimate both CFTR ion-channel function and the protein's proximity to the membrane. We monitor F508del-CFTR, the most common CF-causing variant, and confirm rescue by low temperature, CFTR-targeting drugs and second-site revertant mutation R1070W. In addition, we characterize a panel of 62 CF-causing mutations. Our measurements correlate well with published data (electrophysiology and biochemistry), further confirming validity of the assay. Finally, we profile effects of acute treatment with approved potentiator drug VX-770 on the rare-mutation panel. Mapping the potentiation profile on CFTR structures raises mechanistic hypotheses on drug action, suggesting that VX-770 might allow an open-channel conformation with an alternative arrangement of domain interfaces. The assay is a valuable tool for investigation of CFTR molecular mechanisms, allowing accurate inferences on gating/permeation. In addition, by providing a two-dimensional characterization of the CFTR protein, it could better inform development of single-drug and precision therapies addressing the root cause of CF disease.

Anion flow mediated by the cystic fibrosis transmembrane conductance regulator (CFTR), an apical epithelial channel (1), controls volume and composition of the luminal fluid comportment in several organs. CFTR function is thus crucial for physiological processes, such as airway mucociliary clearance, secretion of pancreatic juices, and maintenance of optimal fluid content in the intestinal lumen (2).

Enterotoxin-induced secretory diarrheas are a major global cause of malnutrition, impaired development and death of children (3). Excessive CFTR-mediated anion conductance (*G*_CFTR_) in the apical membrane of enterocytes causes intestinal loss of large volumes of fluid, leading to dehydration (4). At the other extreme, cystic fibrosis (CF) a common life-limiting genetic disease (5), is caused by mutations that reduce *G*_CFTR_ throughout the body, severely impacting life expectation and quality (6, 7).

*G*_CFTR_ is the product of three factors: the number of channels in the relevant membrane (*N*), channel open probability (*P*_o_), and single-channel conductance (γ),

(Eq. 1) $$G_{CFTR}=N\cdot P_{o}\cdot \gamma$$

Mutations and bacterial toxins can affect gating and permeation of the mature channel (affecting *P*_o_ and γ, respectively). But biogenesis of polytopic CFTR is complex (8, 9), and many mutations (and chemical compounds (10)) also impair folding, trafficking, and plasma membrane stability, resulting in a smaller number of channels at the membrane (*N*).

Drugs targeting CFTR are emerging: CFTR inhibitors, which could provide emergency treatment for diarrheas (11),^3^ and CFTR modulators, capable of restoring CFTR activity to defective mutant channels for CF treatment. Modulators belong to two classes: “potentiators” increase *P*_o_, whereas “correctors” increase plasma membrane density. The potentiator ivacaftor (VX-770, Vertex Pharmaceuticals (12)) dramatically improves lung function of patients carrying G551D (13) or other mutations impairing channel function. Treatment with corrector VX-809 (14), in combination with VX-770, slightly but significantly improves the health of patients carrying two copies of the very common F508del-CFTR variant (15). New triple combination therapies, combining two different correctors with VX-770 further broadened the reach of modulator treatment, with recent demonstration of clinical benefits for patients carrying at least one copy of F508del-CFTR (covering ∼90% of the CF population currently in the CFTR2 database, RRID:SCR_019078) (16, 17).

Despite these major clinical success stories, little is known about how modulators work. An atomic-resolution structure of a VX-770–bound CFTR (18) reveals the superficial binding of the drug molecule at the interface between the transmembrane domain and lipid bilayer. But the binding of the drug is not seen to cause any significant conformational change (compare PDB entry 6O2P, VX-770–bound (18) *versus* PDB entry 6MSM, drug-free (19)), and the permeation pathway remains closed (18, 19). How does VX-770 binding increase *P*_o_ of WT-CFTR and many mutant CFTR versions?

To investigate questions such as these and test mechanistic hypotheses, an assay that allows rapid functional screening of changes caused by mutations or compound modification would be useful. But currently available (relatively high-throughput) assays report on either CFTR surface expression (*e.g.* see Refs. 20 and 21) or CFTR-mediated cellular conductance (22). Apart from low-throughput single-channel patch-clamp recording, assays that measure CFTR function cannot simultaneously measure how many channels are contributing to such function. They cannot discriminate whether a measured conductance arises from a small number of channels with high (*P*_o_·γ) or a larger number of channels with less favorable gating/permeation characteristics.

Here, we present a “high-content” assay based on dual-color live imaging of HEK293 cells that extracts information on both key characteristics of CFTR: by co-expressing soluble mCherry with the halide-sensitive YFP (23) linked to CFTR (24), our new assay gives simultaneous estimates of both CFTR function and CFTR membrane proximity. Experimental manipulations—incubation at low temperature (25, 26, 27), treatment with VX-809 (28, 29) with and without VX-770 (30, 31), and the addition of revertant mutation R1070W (29, 32, 33)—result in the expected changes in measured F508del-CFTR channel function and membrane proximity. In addition, we present a screening platform suitable for describing the molecular characteristics of 62 missense CFTR variants carried by CF patients, and we profile the effects of VX-770 on this panel. Measurements that we obtain correlate well with published data sets, validating our assay as a new tool to investigate questions on CFTR molecular mechanisms and pharmacology.

## Results

### The assay

##### Ion-channel function

Expression of a cytosolic halide-sensitive YFP with increased affinity for iodide and a low affinity for chloride, YFP(H148Q/I152L) (23, 34), allowed the first high-throughput CFTR screening projects, assessing CFTR activity by measuring the rate of YFP fluorescence quenching caused by iodide/chloride exchange across the plasma membrane (35, 36, 37, 38). To obtain quantitative information about ion-channel function, we fused this YFP to the intracellular N-terminal of CFTR (24, 39). We constructed the pIRES2-mCherry-YFPCFTR plasmid that directs co-expression of YFP(H148Q/I152L)-CFTR (hereafter designated YFP-WT-CFTR or simply WT-CFTR) and a soluble, cytosolic, red fluorescent protein, mCherry (40), with both coding sequences transcribed on a single bicistronic mRNA. HEK293 cells are transiently transfected, and images are automatically acquired (before and after iodide addition) and analyzed. The time course of YFP quenching in response to extracellular iodide addition informs on anion conductance. Thanks to the common mRNA, mCherry expression serves as an internal standard for the normalization of YFP-CFTR expression, reducing variability due to unequal transfection efficiency.

##### Membrane proximity

**Figure 1:**
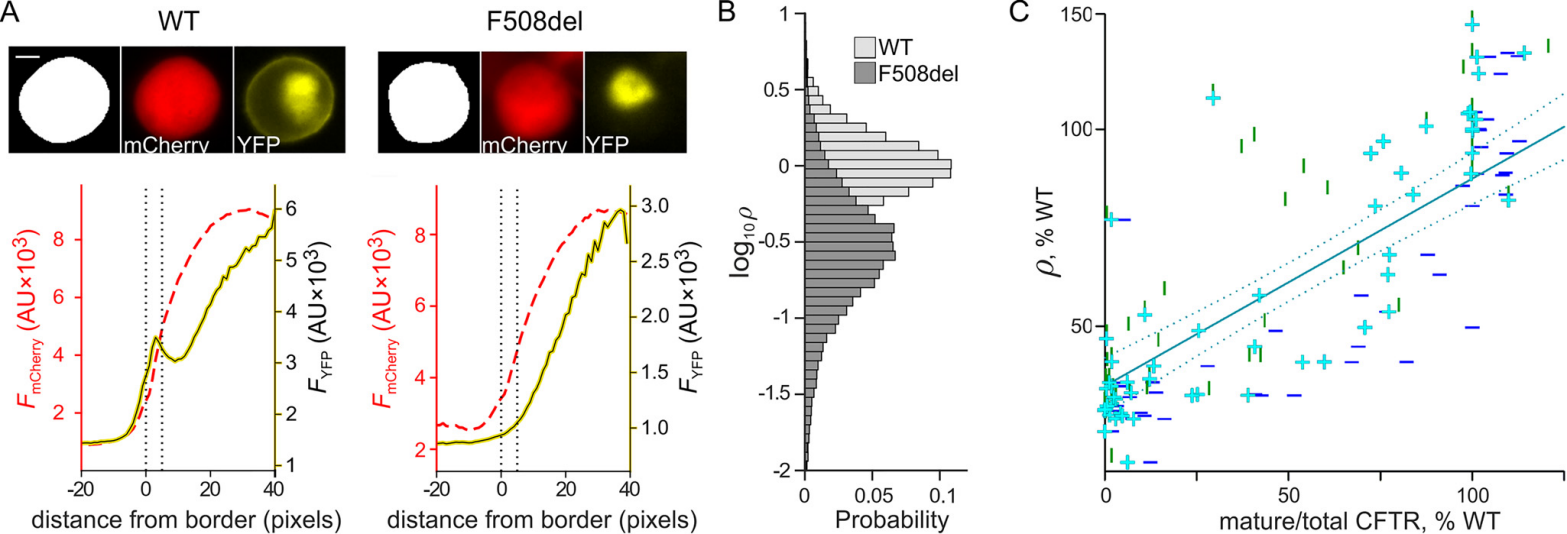
**Quantifying CFTR membrane proximity.***A*, image analysis of individual representative HEK293 cells transfected with pIRES2-mCherry-YFP-WT-CFTR (*left*) and pIRES2-mCherry-YFP-F508del-CFTR (*right*). *Top panels*, boundary delimiting cell (*white*) from non-cell (*black*) is obtained from mCherry image (*center*). CFTR cellular localization is obtained from YFP image (*right*). *Scale bar*, 5 μm. *Bottom panels*, average mCherry fluorescence intensity (*F*_mCherry_, *red dashed line*; *AU*, arbitrary units) and average YFP fluorescence intensity (*F*_YFP_, *solid yellow line*), as a function of the distance from cell border. Membrane proximity, ρ, is defined as $\rho =\frac{F_{\text{YFP}\text{membrane}}}{F_{\text{mCherry cell}}}$, where *F*_YFP membrane_ is the average fluorescence intensity within the “membrane-proximal zone,” set between 0 and 5 pixels from the cell border (*vertical dotted lines*). For the representative cells shown, ρ = 1.60 (WT) and 0.25 (F508del). *B*, probability distribution of log_10_ρ for cells expressing YFP-WT-CFTR (*light gray*) and YFP-F508del-CFTR (*dark gray*), incubated at 37 °C. *C*, correlation between the ρ metric and published data on complex glycosylation. The latter were obtained from quantifying the ratio C-band/(C-band + B-band) in Western blots, from FRT cell lines stably expressing missense mutation CFTR variants. *Vertical green lines* relate our rare-mutation panel with data from Refs. 41 and 43 (*r*^2^ = 0.53); *horizontal blue lines* with data from Ref. 42 (*r*^2^ = 0.74); *cyan plus signs* with averaged values from the latter two data sets (*r*^2^ = 0.67). *Solid* and *dotted cyan lines* are the regression line and 95% confidence intervals, respectively, for the average data set. There are no error bars in Figure 1.

mCherry expression also allows image segmentation and accurate localization of the cell membrane by marking the border of cells. The “membrane-proximal zone” is defined as comprising a ∼1-μm-wide band, on the inside of a cell's boundary (Fig. 1*A*). To obtain a robust relative estimate of the number of channels (*N*) giving rise to the *G*_CFTR_, we estimate overall “CFTR membrane proximity” in each cell calculating the metric ρ. This is obtained by dividing the average YFP-CFTR fluorescence intensity within the membrane-proximal zone (*F*_YFP membrane_) by the average mCherry fluorescence over the entire cell (*F*_mCherry cell_). The ρ metric can be thought of as the product of the *F*_YFP membrane_/*F*_YFP cell_ metric, the proportion of YFP-CFTR within the membrane-proximal zone, multiplied by the metabolic stability of YFP-CFTR with respect to mCherry (*F*_YFP cell_/*F*_mCherry cell_). Thus, changes in the ρ metric will reflect not only changes in efficiency of CFTR maturation and trafficking, but also changes in the overall rates of biosynthesis *versus* degradation of the protein.

The distribution of ρ measurements, easily obtained for hundreds of cells in individual images, is skewed but approaches a log-normal distribution. Values were log-transformed (Fig. 1*B*) before performing statistical analysis.

The ρ metric is related to a commonly used measure of CFTR biogenesis, the proportion of protein acquiring complex glycosylation (*i.e.* that has undergone Golgi processing), estimated using protein blotting. For a set of CF-causing missense mutations (see rare-mutation panel, below), we found a good correlation (*r*^2^ = 0.67) of our ρ measurements with published data sets (41, 42, 43) (Fig. 1*C*; see also Fig. S1). Note that methodologies and materials used were very different: fluorescence measurements in transiently expressing HEK293 cells *versus* Western blots from stably expressing Fischer rat thyroid, (FRT) cell lines.

For both methodologies, CFTR proteins located in post-Golgi, submembrane compartments cannot be discriminated from those at the plasma membrane, directly contributing to *G*_CFTR_. Nevertheless, both measurements, by detecting defects in processing and metabolic stability, provide useful rough estimates of relative plasma membrane numbers.

### Rescue of F508del-CFTR membrane proximity

**Figure 2:**
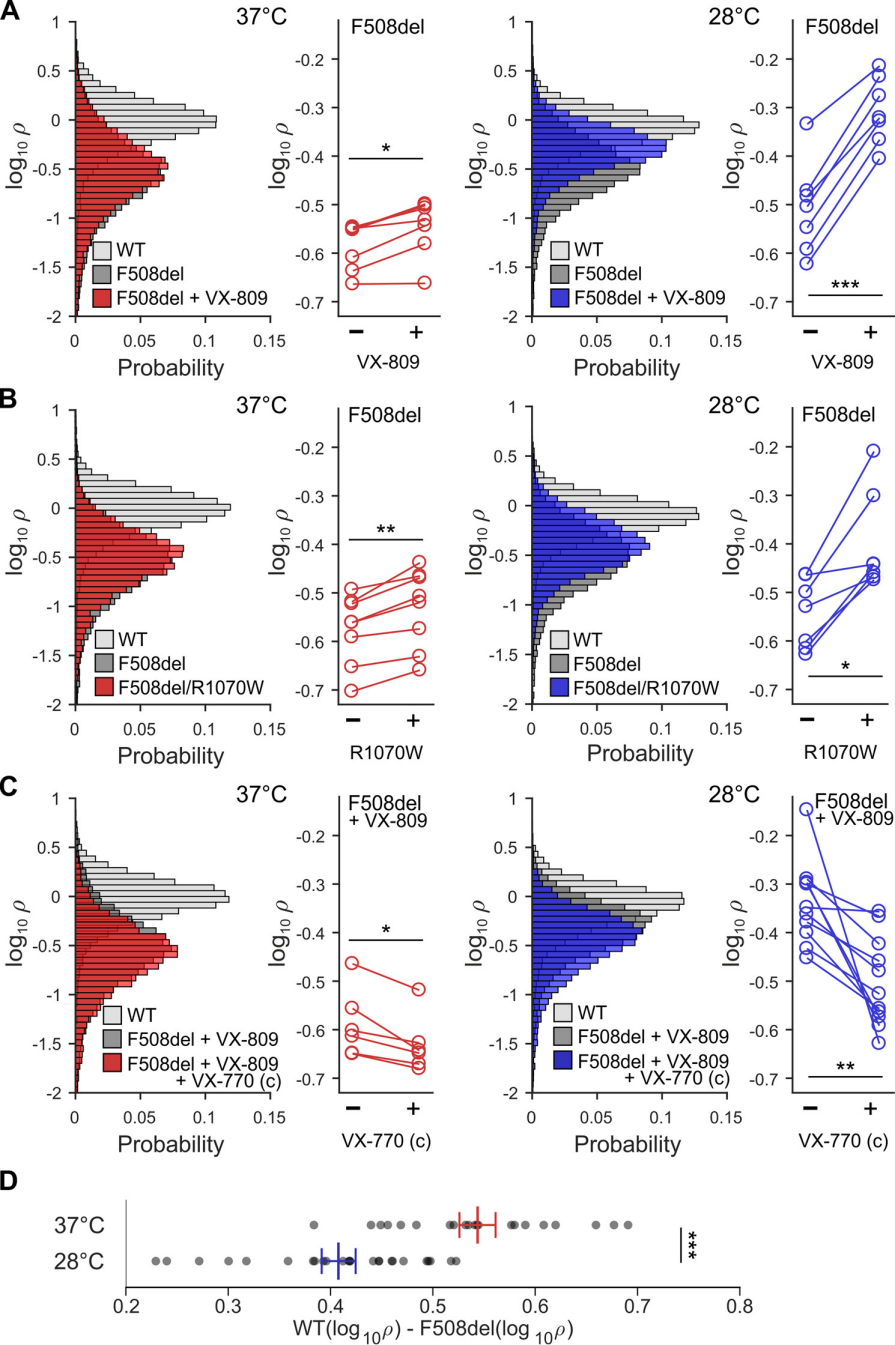
**Quantifying rescue of F508del-CFTR membrane proximity.** Effects of chronic treatment with 10 μm VX-809 (*A*), R1070W rescue (*B*), and chronic treatment with 10 μm VX-809 ± 10 μm VX-770 (C), on log_10_ρ at 37 °C (*left*, *red*) and 28 °C (*right*, *blue*). Conditions of final incubation were maintained during image acquisition. The probability distributions in the *panels* on the *left* contain log_10_ρ measurements from thousands of cells, pooled from all experiments. For statistical analysis, mean log_10_ρ values determined in independent experiments (individual 96-well plates), and paired per plate, were used (displayed in *panels* on the *right*, *line* connecting measurements from the same plate. *, *p* < 0.05; **, *p* < 0.01; ***, *p* < 0.001, adjusted *p* values after Bonferroni correction). *D*, before imaging, plates were incubated at 37 or 28 °C for 24 h. For each plate, the difference between mean log_10_ρ for WT-CFTR and F508del-CFTR was calculated (WT(log_10_ρ) − F508del(log_10_ρ); *gray dots*). *Red* (37 °C) and *blue* (28 °C) *lines* show mean ± S.E.M., calculated from 21 (37 °C) and 25 (28 °C) within-plate difference estimates. *Error bars*, S.E.M.

As a first validation of our assay, we assessed changes in F508del-CFTR membrane proximity by comparing distributions of log_10_ρ (logarithmic transformation of the ρ metric) following treatments/mutations known to partially rescue the F508del processing defect (Fig. 2).

##### F508del-CFTR membrane proximity rescue by VX-809 incubation

At 37 °C, incubation with corrector drug VX-809 for 24 h caused a very small, but significant, increase in log_10_ρ of F508del-CFTR, (Fig. 2*A* (*left*); see also Table S1). At 28 °C, the magnitude of the increase was greater (Fig. 2*A*, *right*).

##### F508del-CFTR membrane proximity rescue by R1070W second-site revertant mutation

Introducing the mutation R1070W, known to partially revert the F508del-CFTR–defective phenotype (33), significantly increased F508del-CFTR membrane proximity at 37 °C (Fig. 2*B* (*left*) and Table S1), as well as at 28 °C (Fig. 2*B* (*right*) and Table S1). Again, the magnitude of the effect was larger at 28 °C.

##### F508del-CFTR membrane proximity decrease due to chronic VX-770 incubation

Chronic incubation with VX-770 has been shown to have a negative impact on VX-809 correction of F508del-CFTR biogenesis (30, 31). When comparing cells expressing F508del-CFTR incubated for 24 h with VX-809 alone with those incubated with both corrector VX-809 and potentiator VX-770, at 37 °C, there was a small but significant decrease in log_10_ρ (Fig. 2*C* (*left*) and Table S1). At 28 °C the decrease was again more pronounced than at 37 °C (Fig. 2*C*, *right*).

##### F508del-CFTR membrane proximity rescue by temperature correction

Temperature could only be varied between plates, preventing the use of within-plate differences in log_10_ρ to directly compare membrane proximity of F508del-CFTR incubated at different temperatures. We therefore compared the magnitude of the within-plate difference between F508del-CFTR and WT-CFTR for plates incubated at 28 and at 37 °C. The log_10_ρ values of F508del-CFTR were significantly closer to those of WT-CFTR at 28 °C than at 37 °C, (Fig. 2*D* and Table S1).

### Rescue of F508del-CFTR ion channel-function

**Figure 3:**
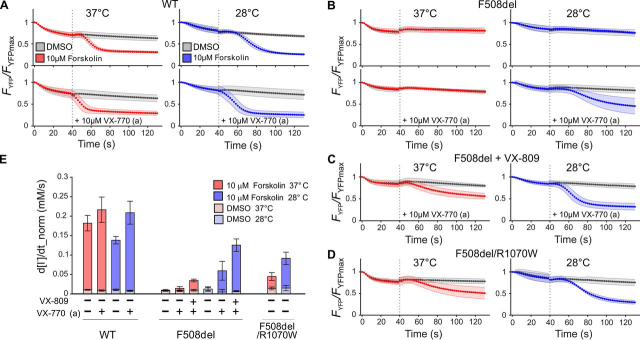
**Rescue of F508del-CFTR ion-channel function.***A–D*, quenching of YFP fluorescence in HEK293 cells expressing WT-CFTR (*A*), cells expressing F508del-CFTR chronically (24 h) treated with vehicle only (DMSO) (*B*) or with VX-809 (*C*), or cells expressing R1070W/F508del-CFTR (DMSO-only chronic treatment) (*D*). *F*_YFP_/*F*_YFPmax_, observed YFP fluorescence, normalized using fluorescence at the time point before I^−^ addition. For more information on statistical analysis, see Tables S2 and S3. Prior to imaging, plates were incubated for 24 h, at 37 °C (*red*) or 28 °C (*blue*). This final incubation temperature was maintained throughout image acquisition. At time point 0 s, I^−^ was added to the extracellular medium. At 40 s (*dotted line*), forskolin and, where indicated, VX-770 (*acute, a*) were added, both to a final concentration of 10 μm. *E*, the maximal rate of I^−^ entry (normalized by using the *F*_mCherry_ within cell, d[I^−^]/dt_norm) is used to summarize CFTR function for genotypes and conditions shown in *A–D*. *Error bars*, S.E.M.

Functional rescue of F508del-CFTR was also measured. In these experiments, CFTR was activated following the addition of extracellular I^−^ (monitoring of nonstationary CFTR activity; see “Experimental procedures”). Activation occurred either by the addition of only 10 μm forskolin, increasing intracellular cAMP and thus CFTR phosphorylation, or by the addition of a combination of 10 μm forskolin and 10 μm VX-770 (the latter defined as an acute (a) treatment, as opposed to the 24-h chronic (c) incubation with VX-770 described above). YFP fluorescence (normalized using the fluorescence reading before the I^−^ addition) was followed over time (*F*_YFP_/*F*_YFPmax_; Fig. 3). The maximal rate of I^−^ entry (Δ[I^−^]_in_/Δ*t*) was used to summarize CFTR channel function. For each different CFTR genotype, incubation, and activation condition tested, the Δ[I^−^]_in_/Δ*t* obtained from quenching time curve analysis was normalized (using the corresponding mean *F*_mCherry_ within the cell selection) to take into account differences in transfection efficiency (d[I^−^]/dt_norm; Fig. 3*E* and Tables S2 and S3). No significant difference in this metric was detected among the different genotypes/conditions when DMSO (vehicle) was added instead of activators.

##### WT-CFTR

Measurements from HEK293 cells expressing WT-CFTR were taken for comparison purposes. As expected, the maximal rate of I^−^ entry was significantly higher after activation with forskolin, compared with control (DMSO), at both 37 and 28 °C (Fig. 3 (*A* and *E*), *WT*, and Table S2). However, conditions were optimized for measuring low CFTR activity, and neither the presence of 10 μm VX-770 in addition to forskolin during activation, nor incubation at 37 °C *versus* 28 °C increased quenching rate sufficiently to achieve statistical significance after multiple comparison correction (Fig. 3 (*A* and *E*), *WT*, and Table S3).

##### F508del-CFTR functional rescue following temperature correction

Following incubation at 28 °C for 24 h, activation with forskolin alone failed to increase the maximal rate of I^−^ entry in untreated cells expressing F508del-CFTR (Fig. 3 (*B* (*top*) and *E* (*F508del bars 1* and *4*) and Table S2), reflecting the severe gating defect, which persists even after temperature correction. Acute potentiation by VX-770 was required to detect function of the channels reaching the cell surface due to temperature correction (Fig. 3 (*B* (*bottom*) and *E* (*F508del bar 5 versus bar 2*) and Table S2).

##### F508del-CFTR functional rescue following VX-809 correction

At both temperatures, acute potentiation revealed the activity of F508del-CFTR channels that had reached the cell surface due to 24-h incubation with VX-809. At 28 °C, the maximal rate of I^−^ entry was significantly greater than at 37 °C (Fig. 3*C* and *E* (*F508del bar 6 versus bar 3*) and Table S3).

##### F508del-CFTR functional rescue by the R1070W mutation

Forskolin activation alone was enough to reveal *F508del/R1070W*-CFTR channel activity (Fig. 3*D* and Table S2). The maximal rate of I^−^ entry was significantly higher at 28 °C than at 37 °C (Fig. 3*D* and *E* (*F508del/R1070W*) and Table S3).

### The rare-mutation panel

More than 300 CF-causing mutations have been characterized (The Clinical and Functional TRanslation of CFTR (CFTR2); available at RRID:SCR_019078). CF-causing missense CFTR mutations (41, 42, 43) were individually introduced in the pIRES2-mCherry-YFPCFTR plasmid, creating a panel of 62 plasmids (including WT-CFTR as reference).

**Figure 4:**
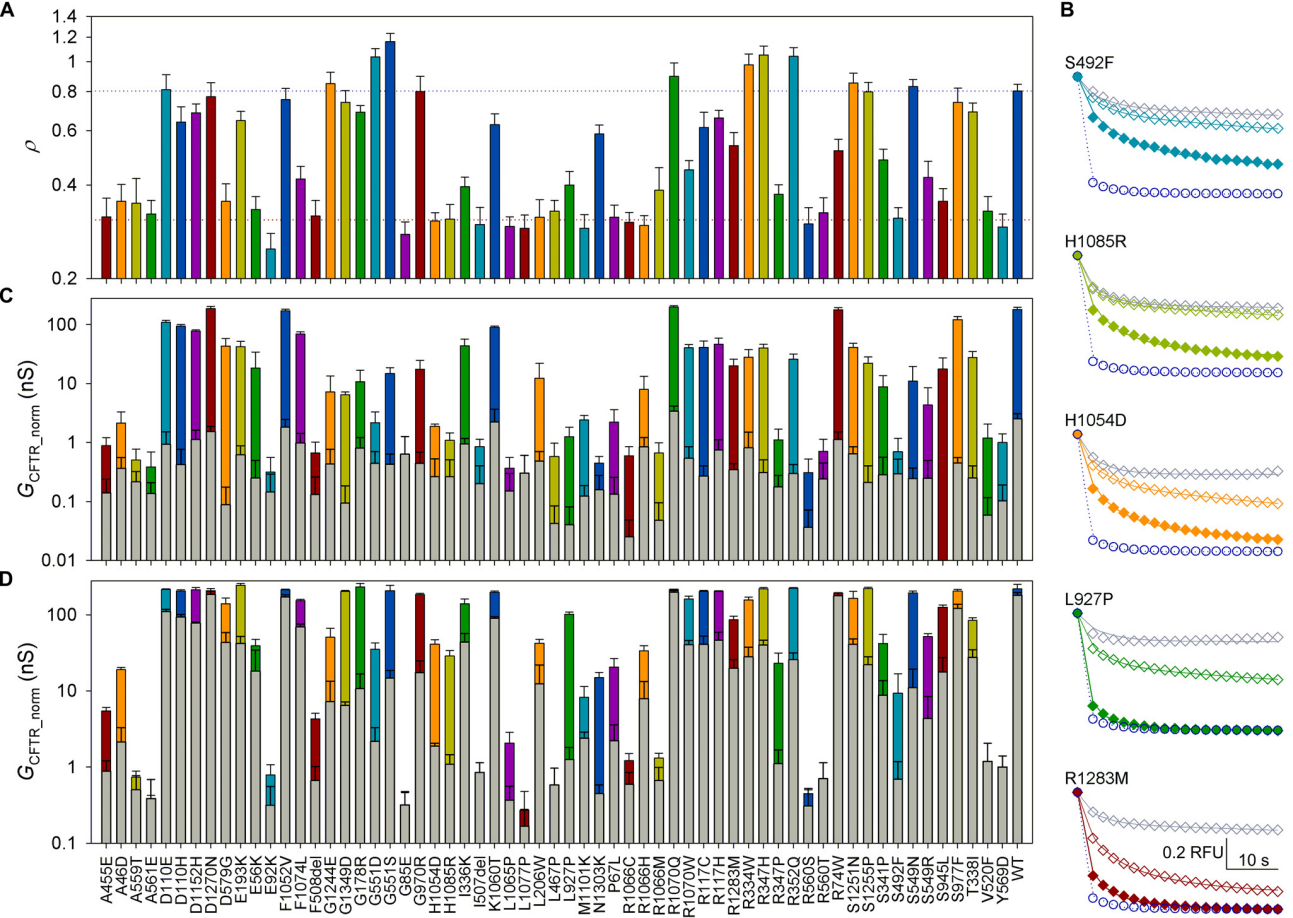
**Rare CF mutation profiling.***A*, mean ρ (*n* ≥ 9) of all mutations in the *panel*. *Blue* and *red dotted lines* indicate mean ρ for WT- and F508del-CFTR, respectively. For ρ distributions and mean ρ and *n* values for each mutant, see Fig. S2 and Table S3. *B*, observed YFP fluorescence quenching time course (*y* axis, measured in relative fluorescence units (*RFU*) is *F*_YFP_/*F*_YFPmax_: observed YFP fluorescence, normalized using fluorescence at the time point before I^−^ addition) after activation with DMSO (*gray diamonds*), 10 μm forskolin (*empty colored diamonds*), or 10 μm forskolin + 10 μm VX-770 (a) (*filled colored diamonds*) for selected mutations. *Solid lines*, predicted change in proportion of anion-free YFP. For estimated parameters *G*_CFTR_, *V*_M_, *G*_trans_, and τ_trans_, see Table S7. WT-CFTR quenching in 10 μm forskolin (*dark blue empty circles*, observed; *dotted line*, fit) is shown for comparison. *C*, CFTR conductance of the rare-mutation panel after activation with 10 μm forskolin (*colored bars*) or vehicle control (DMSO, *gray bars*). *n* ≥ 3. *G*_CFTR_, obtained from fitting of the quenching time course for each mutant (as shown in *B*), was normalized using the mean within-cell mCherry fluorescence for that mutant, measured with respect to the corresponding metric obtained for WT-CFTR on the same plate. For statistical analysis, see Table S5. *D*, potentiation of rare-mutation panel by VX-770. *Gray bars*, values following activation with 10 μm forskolin alone; *colored bars*, values with the further addition of 10 μm VX-770 (a). For statistical analysis, see Table S6. *Error bars*, S.E.M.

Following expression of the panel in HEK293 cells and incubation with no pharmacological correction, distributions for the ρ metric, and plate log_10_ρ means were obtained (Table S4 and Fig. S2). The data are summarized in Fig. 4*A*, which profiles membrane proximity for each CFTR mutant variant in the panel.

As mentioned above, correlation between our measured ρ and the proportion of CFTR protein acquiring complex glycosylation in FRT cells is very good (*r*^2^ = 0.74 (42), *r*^2^ = 0.53 (41, 43), and *r*^2^ = 0.67 using average values for mutants measured by both groups (41, 42, 43) (Fig. 1*C* and Fig. S1).

The time course of YFP fluorescence quenching was also acquired and analyzed (see “Quantification of CFTR activity at steady state”). In these experiments, steady-state *G*_CFTR_ was estimated from the *F*_YFP_/*F*_YFPmax_ time curve and then normalized using the within-cell *F*_mCherry_, to yield *G*_CFTR_norm_. For each genotype, quenching was monitored with no activation (DMSO) or following baseline preactivation with 10 μm forskolin (Fig. 4 (*B* and *C*) and Table S5). Again, results correlate well with published data (*r*^2^ = 0.68 (42), *r*^2^ = 0.61 (41, 43), *r*^2^ = 0.60 (41, 42, 43); Fig. S1). Conductance was also measured following preactivation with 10 μm forskolin + 10 μm VX-770 (a) (Fig. 4 (*B* and *D*) and Table S6). In these conditions, genotypes with high conductance (including WT-CFTR) have faster YFP quenching than can be reliably measured in our system. However, the assay can accurately monitor VX-770 potentiation when CFTR activity is low, as is the case for most mutants.

### Relationship between CFTR ion-channel function and membrane proximity

By considering changes in ion-channel function in the context of any change measured in ρ, our assay allows accurate inferences on the gating and permeation properties of the CFTR channel molecules present at the cell surface.

**Figure 5:**
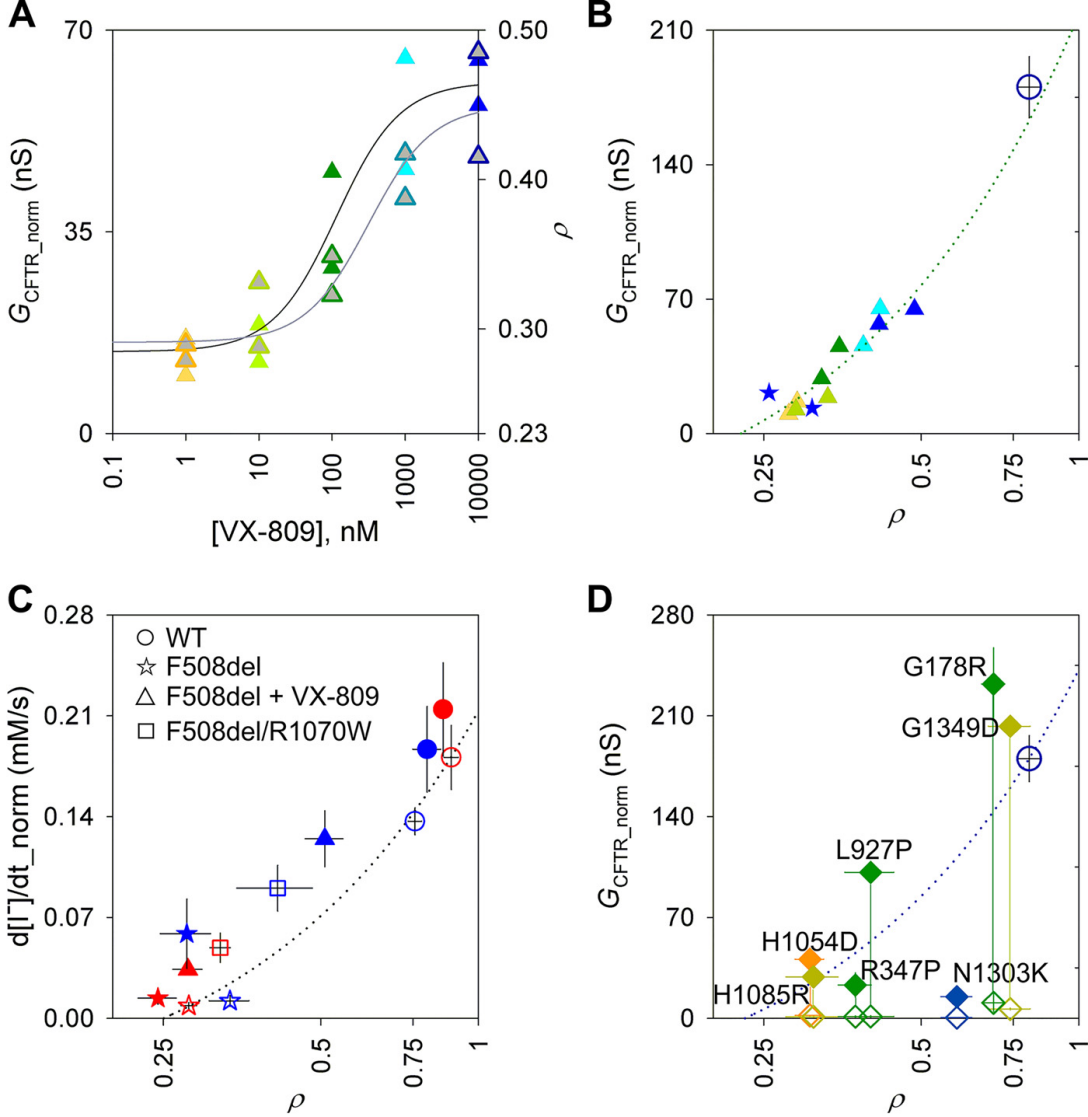
**Inferring permeation/gating characteristics.***A*, dose-response plot of increase in conductance (*left axis*, *colored symbols*, *black fit line*) and membrane proximity (*right axis*, *gray-filled symbols*, *gray fit line*) following incubation of F508del-CFTR with increasing concentrations of VX-809. *Lines* represent fits to the Hill equation (four parameters, *n*_H_ constrained to 1; see Ref. 24). Only two measurements were taken at each concentration, but the EC_50_ values we obtain (114 ± 66 and 316 ± 238 nm for G_CFTR_ and ρ, respectively) are not dissimilar from published values (14, 21). *B*, relationship between normalized CFTR conductance and membrane proximity in cells expressing F508del-CFTR with no correction (*blue stars*) or incubated with increasing concentrations of VX-809 (1 nm to 10 μm, *color-coded* as in *A*), all after activation with 10 μm forskolin and 10 μm VX-770 (a). F508del-CFTR incubation and measurements were at 28 °C. *Green dotted line* shows linear regression using only F508del-CFTR data points on the graph (slope = 281.7, constant = −63.7, resulting in an *x* axis intercept at ρ = 0.23). The mean value for WT-CFTR activated with 10 μm forskolin alone (*large dark blue empty circle*) is shown for reference (from *D*; see also Fig. 6). *C*, relationship between maximal rate of I^−^ influx and ρ in HEK293 cells expressing WT-CFTR, F508del-CFTR, and F508del/R1070W-CFTR, at 37 °C (*red symbols*) and 28 °C (*blue symbols*). *Empty symbols* indicate CFTR activation with 10 μm forskolin alone; *solid symbols* indicate further acute potentiation with 10 μm VX-770. *Dotted line*, linear interpolation between data obtained at 37 °C for uncorrected F508del-CFTR (used as an empirical measure of minimal membrane proximity) and WT-CFTR, both without acute VX-770 potentiation; slope = 0.284, constant = −0.071, resulting in an *x* axis intercept at ρ = 0.25. *D*, mutants with the largest -fold potentiation by VX-770 (ratio between conductance obtained in 10 μm forskolin + 10 μm VX-770 (a) over that in 10 μm forskolin alone is >20). *Empty diamonds*, baseline activation with 10 μm forskolin alone; *solid diamonds*, activation following acute potentiation with 10 μm forskolin + 10 μm VX-770 (a). *Error bars*, S.E.M.

Even when virtually no channels are present in the plasma membrane (as happens, for instance, for cells expressing F508del-CFTR grown at 37° C), the value of ρ does not fall to zero. This is likely due to some inaccuracy in automated cell boundary delineation and to the widefield microscope optics, resulting in stray light from out-of-focus planes reaching the photomultiplier. To empirically investigate the relationship between *G*_CFTR_ and ρ, cells expressing F508del-CFTR (temperature-corrected and acutely potentiated with VX-770 to maximize small signals) were treated with increasing concentrations of corrector VX-809, progressively improving both biogenesis/membrane stability and conductance (Fig. 5, *A* and *B*). Measured *G*_CFTR_norm_ values as a function of ρ values show a roughly linear relationship (Fig. 5*B*, *dotted green line*). The line can be extended to cross the ρ axis, extrapolating to an intercept at ρ = 0.23. In addition, in as much as ρ values are proportional to the number of channels at the membrane (*N*), the steepness of this line is an estimate of the product (*P*_o_·γ). An extension of the line toward higher membrane proximity values shows the *G*_CFTR_norm_ values expected with a higher number of channels reaching the membrane but retaining gating/permeation characteristics of F508del-CFTR, acutely potentiated by VX-770. It can be seen that, in these conditions, F508del-CFTR is characterized by *P*_o_ levels similar to those of WT-CFTR (the latter without potentiation; Fig. 5*B*, *large dark blue empty circle*, *not far above dotted green line*), consistent with patch-clamp measurements (note that γ is unaffected by the F508del mutation) (44, 45).

Data on the maximum rate of I^−^ entry can also be plotted against the corresponding ρ values, measured for the different F508del-CFTR rescue strategies (Fig. 5*C*). A linear interpolation between data points for uncorrected F508del-CFTR at 37 °C (representing cells with virtually no CFTR molecules at the membrane) and WT-CFTR activated by 10 μm forskolin at 37 °C describes the ion-channel function we would expect from cells with increasing CFTR membrane proximity, assuming gating and permeation characteristics of baseline-activated WT-CFTR (Fig. 5*C*, *blue dotted line*). This allows us to infer how the rescued F508del-CFTR channels reaching the membrane compare with control channels in terms of permeation/gating.

Introducing the R1070W revertant mutation in the F508del-CFTR background is shown to be particularly effective in improving gating (note that permeation and single-channel conductance are unaffected by both F508del and R1070W mutations (33, 46)). R1070W revertant rescue and temperature correction similarly increase membrane proximity. However, temperature-corrected F508del-CFTR channels at the membrane have very low ion-channel function (unless acutely potentiated with VX-770). In contrast, F508del/R1070W channels at the membrane have gating and permeation properties equal—or even superior—to WT-CFTR (Fig. 5*C*, *cf*. uncorrected F508del-CFTR *blue star symbol versus* F508del/R1070W-CFTR *red square symbol*, both compared with *blue dotted line*). Both results are consistent with patch-clamp records indicating a F508del/R1070W-CFTR *P*_o_ comparable with that of WT-CFTR (47) but a much lower *P*_o_ for temperature-corrected F508del-CFTR (44, 45, 47).

**Figure 6:**
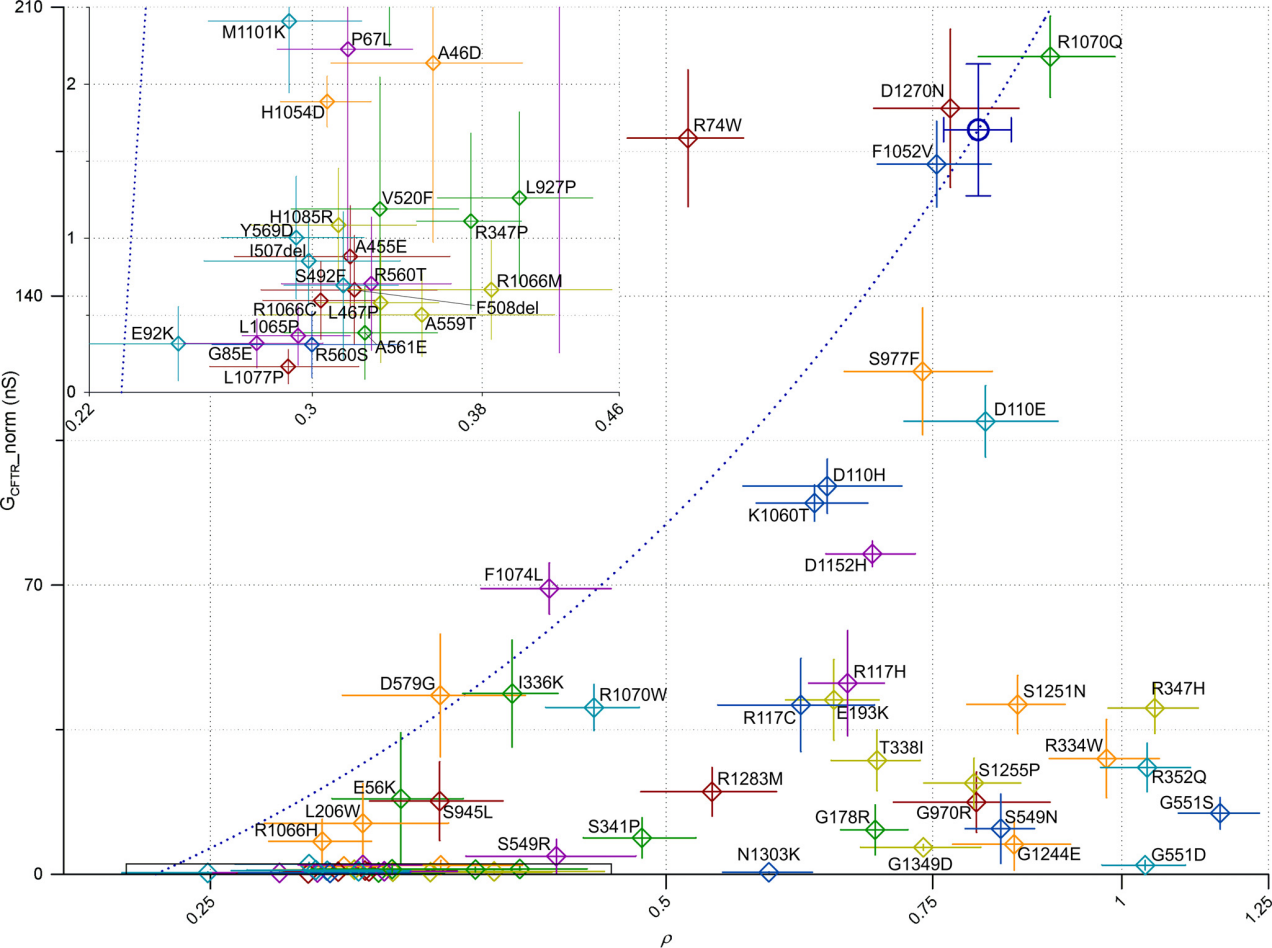
**Relationship between baseline *G*_CFTR__norm (10 μm forskolin) and ρ for the rare-mutation panel.***Colors* are as in Fig. 4. WT-CFTR is highlighted as a *large*, *dark blue*, *empty circle*. The *dark blue dotted line* (slope = 314.1, constant = −72.3) shows linear interpolation between WT data point and *x* axis intercept set at ρ = 0.23, as obtained in Fig. 5*B*. *Inset*, *expanded axes view* of area indicated by the *black rectangular outline* (0 < G_CFTR__norm < 2.5 nS; 0.22 < ρ < 0.46). *Error bars*, S.E.M.

Fig. 6 plots *G*_CFTR_ as a function of ρ for the rare-mutation panel, giving an immediate representation of how severe a defect each mutation causes in biogenesis (distance from WT-CFTR on the *x* axis) and/or in gating and permeation properties (vertical displacement from *blue dotted line*, which assumes ion-channel properties of baseline-activated WT-CFTR). For instance, D579G-CFTR (*orange open diamond* at coordinates (0.35,41.5)) falls close to the WT-CFTR line, suggesting that the product (*P*_o_·γ) is not greatly affected by this mutation and that the low short-circuit currents measured in FRT cells (41, 42) are largely caused by the reduced membrane density. For G1244E (*orange* (0.85,7.2)) and S549N (*blue* (0.83,11)), likely altering the structure of CFTR's canonical ATP-binding site 2 (in the P-loop and signature sequence loop, respectively), measured ion-channel function is much lower than would be expected given the high observed membrane proximity. Here, low short-circuit currents (42) are likely due to gating defects. Most mutations give reduced membrane proximity and a conductance that falls below the WT interpolation line, suggesting processing defects as well as some degree of impairment in gating/permeation for the CFTR molecules that do reach the membrane. We further illustrate the effect of acute treatment with VX-770 for mutations resulting in the strongest potentiation (-fold potentiation >20; Fig. 5*D*). For many of these, data points for potentiated conductance fall *above* the interpolation line, suggesting that the product (*P*_o_·γ) is higher than measured for WT-CFTR in baseline-activated conditions.

## Discussion

The results presented in this paper introduce and validate a new fluorescence assay for monitoring the CFTR protein. To monitor ion-channel function, it exploits a previously described YFP-CFTR fusion (24, 39). The N-terminal fusion of a fluorescent protein has been shown to cause only very minor alterations to CFTR's biogenesis and function (24, 39, 48, 49, 50, 51). In addition, the new assay provides a simultaneous estimate of CFTR biogenesis, quantified in terms of membrane proximity.

### Validation of the assay

##### Validation of membrane proximity measurements

Although heterogeneity among ρ values for individual cells is large, resulting in broad distributions (Fig. 2), much of the variability is related to between-plate variation, such that paired comparisons between measurements obtained in the same plate (*right panels* in Fig. 2) can pick up small changes in membrane proximity, increasing assay sensitivity.

For instance, we measure small changes in F508del-CFTR membrane proximity due to incubation with corrector VX-809 at 37 °C. Whereas one published paper reports large effects of this corrector, resulting in rescue of up to 15% of WT-CFTR function (14), much more limited effects are measured by other groups (a 3–4-fold increase in plasma membrane density or function, starting from a value of ∼1% of WT (29, 52)). Our assay detects a change in membrane proximity of a similar magnitude to the latter reports (*cf.* Refs. 29 and 52 *versus* Fig. 2*A* (*left*)). These limited *in vitro* effects are more in agreement with the inability of VX-809 monotherapy to improve lung function for F508del homozygous patients (53).

The effect we measure for the R1070W mutation at 37 °C is similarly small but also significant (Fig. 2*B*, *left*). Again, our result confirms observations published by others: the rescue of membrane-exposed F508del-CFTR due to the R1070W mutation is limited (from 2 to 7% of WT-CFTR), becoming more obvious only when combined with other rescue maneuvers, such as additional revertant mutations or correctors (29).

We could also confirm previous reports demonstrating increased membrane proximity of F508del-CFTR due to low-temperature incubation (25, 26, 27) (Fig. 2*D*) and enhanced effects of VX-809 treatment when combined with incubation at low temperature (28) (Fig. 2*A*, *right*). We further demonstrate that low-temperature incubation also enhances R1070W rescue. The synergy between effects of low temperature and the R1070W mutation, and of low temperature and VX-809 incubation, suggests that, whereas VX-809 and the R1070W mutation are acting via a common mechanism stabilizing the NBD1/TMD interface (between nucleotide binding domain 1 and transmembrane domain) (29), a different pathway, possibly involving proteostasis components (27), likely underlies rescue by low-temperature incubation.

In agreement with other studies (30, 31, 54), we observed a small but significant shift in log_10_ρ following chronic incubation with VX-770, consistent with the potentiator destabilizing F508del-CFTR at the membrane (Fig. 2*C*, *left*). Furthermore, we find that the negative effect of VX-770 on biogenesis appears more pronounced when cells are incubated at 28 °C (Fig. 2*C*). It is possible that binding of VX-770 prevents interaction with chaperone(s) that help F508del-CFTR fold and exit the ER in cells grown at low temperature (27). However, the concentration of VX-770 we used (10 μm) is relatively high (55). Despite the fact that in our incubation medium, as in plasma, a large proportion of the drug will be bound to proteins present in the added serum (56), VX-770 will likely accumulate in the hydrophobic membranes (55, 56). Hence, it is also possible that some of the F508del-CFTR destabilization we observe might be linked to formation of precipitates within cellular membranes (55), which would be more pronounced at the lower temperature.

##### The HEK293 expression system

We implemented our assay in the HEK293 heterologous expression system, characterized by robustness, ease of culture and of genetic manipulation. Whereas HEK293 cells do not form monolayers suitable for functional measurements of transepithelial currents, they are widely used in the study of both CFTR function and biogenesis (57, 58, 59, 60, 61, 62). Our measurements of temperature–, VX-809–, and R1070W–dependent recue of F508del-CFTR membrane proximity (Fig. 2), confirm results obtained using other systems, including human bronchial epithelia (29, 52). In addition, our membrane proximity measurements for the rare-mutation panel (Fig. 4*A*) correlate well (Fig. 1*C* and Fig. S1) with immunoblot measurements obtained with FRT cell lines stably expressing CFTR variants (41, 42), a system known to have *in vivo* predictive value for CF (42, 52). Our study thus validates the use of HEK293 cells as a tool for the molecular characterization of the CFTR protein, including its biogenesis.

However, whereas acute potentiator action is largely independent of the cell system used for testing (*e.g.* VX-770 is effective in a range of expression systems, from *Xenopus laevis* oocytes (55), to primary human bronchial epithelia (63)), there is evidence that CFTR correction involves biosynthetic pathway and quality control components that are cell type–specific (64). Immortalized overexpressing cell lines, even those derived from human bronchial epithelia, do not always predict drug activity in primary cultures for corrector compounds (21). Thus, especially when addressing questions focusing on biogenesis with potential translational impact, studies using our assay will need to be complemented and confirmed by research using material better recapitulating *in vivo* cellular processing. This has been the approach followed for the currently approved correctors VX-809 and VX-661, modifications of hits first identified using an overexpressing mouse fibroblast cell line (65).

##### Accurate measurements of low CFTR ion-channel function

In addition to membrane proximity, our assay quantifies channel function. Here we confirm previously published data, showing how two different protocols—one measuring the maximal rate of I^−^ entry (Δ[I^−^]_in_/Δ*t*) during CFTR activation (24) and the other estimating CFTR conductance by fitting quenching time course after steady-state activation is reached (39)—provide results that are consistent with those obtained with other techniques (*e.g.* Ussing chamber short-circuit current measurements, high-throughput electrophysiology). Thus, both *G*_CFTR_ (Fig. 4 (*B–D*) and Fig. S1) (41, 42, 43) and (Δ[I^−^]_in_/Δ*t*) (Figure 3, Figure 4, Figure 5*B*) (22) can accurately estimate CFTR ion-channel function. In this study, the assay conditions were not optimized to measure high CFTR activities, and some measurements hit the upper limit of its dynamic range (*e.g.* for WT-CFTR; Figure 3, Figure 4 and Table S3). If needed, conditions can be altered to avoid assay saturation (*e.g.* by using lower concentrations of forskolin or [I^−^]_out_).

Accurate quantification of low conductance values is advantageous in characterizing drug response by CFTR mutants that have particularly low residual activity. For instance, our assay detects strong VX-770 potentiation for R347P-, N1303K-, and H1085R-CFTR (Figs. 4*D* and 5*D*), genotypes giving no significant potentiation over baseline in a Vertex Pharmaceuticals study to profile VX-770 sensitivity (41). Our results on N1303K are consistent with patch-clamp and other short-circuit current measurements demonstrating effective potentiation of N1303K-CFTR by VX-770 (66, 67, 68). Despite short-circuit current in FRT cells being increased only to less than the 5% of WT-CFTR threshold (41), caution is required in classifying such mutants as “unresponsive” to VX-770, as they might benefit from therapies combining VX-770 with other modulators (67, 68). Equally promising for possible studies on synergistic modulator effects are L927P- and H1045D-CFTR channels, which, because of very low baseline levels, give potentiated short-circuit currents only slightly above the 5% of WT-CFTR threshold (41) but are also powerfully potentiated (Figs. 4*D* and 5*D*).

### Considerations on VX-770 mechanism of action

**Figure 7:**
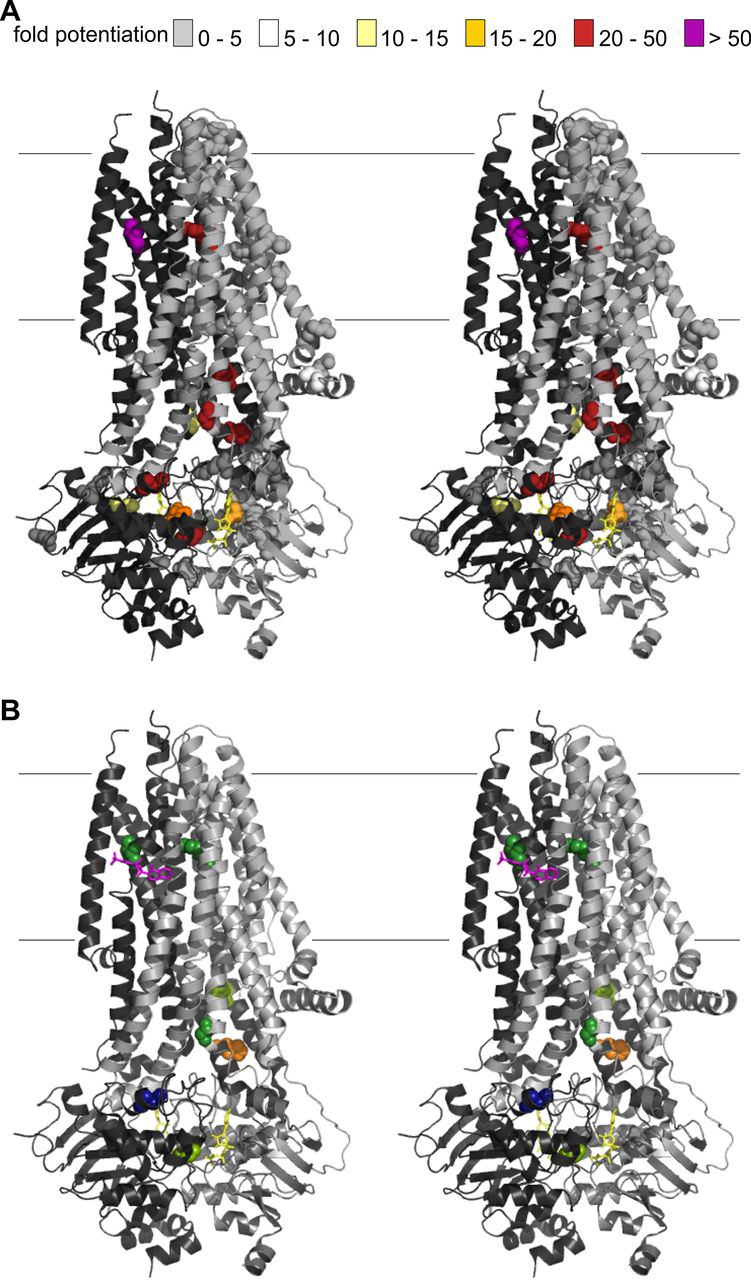
**Mapping VX-770 sensitivity on cryo-EM structures.***A*, *cartoon representation* (*cross-eyed stereo*) of phosphorylated, ATP-bound human CFTR (6MSM (19)), with atoms of missense mutations included in the panel highlighted as *spheres*. *Colors* indicate the degree of -fold potentiation by VX-770. *Light gray*, TMD1-NBD1; *dark gray*, TMD2-NBD2. *Fine horizontal lines* show approximate position of the membrane boundary. *B*, only missense mutation sites with the most efficacious VX-770 potentiation are shown. *Magenta sticks*, position of bound VX-770 in 6O2P structure (18). In a *cartoon representation*, 6O2P and 6MSM are virtually identical (root mean square deviation = 0.14 Å (18). Mutation-site residues are *color-coded* as in Fig. 4 (moving from the cytosol to extracellular): Gly-1349 (*light green*), Asn-1303 (*dark blue*), His-1054 (*orange*), Gly-178 (*forest green*), His-1085 (*light green*), Arg-347 (*forest right*), and Leu-927 (*forest left*).

Our empirical profiling of the VX-770 response in the rare-mutation panel can generate hypotheses on mechanism of action. Focusing on the mutations resulting in the highest VX-770 efficacy (-fold potentiation >20; Figs. 5*D* and 7), we note they can be broadly classified into two groups.

Five of these mutations are part of the ball-and-socket joints (69) linking TMDs to NBDs or located at the NBD1/NBD2 interface. They all introduce charged side chains that would strongly destabilize an NBD-dimerized open-state conformation: the introduced charges would be interacting unfavorably with other close charges (α-carbon distances <10 Å, in PDB entry 6MSM structure of phosphorylated, ATP-bound human CFTR carrying the open state–stabilizing E1371Q mutation (19); see Table S8). A destabilization of the ABC-canonical, NBD-dimerized, open-channel conformation (70) is thus likely the cause of the low conductance measured after baseline activation in these mutants. Consistent with this interpretation, N1303K-CFTR channels appear to have almost completely lost the coupling between NBDs and TMDs that normally controls gating, and the rare openings observed are not linked to ATPase cycles at the NBDs (68). However, for all of these mutant channels, conductance is greatly increased by VX-770. Thus, the VX-770–bound open state must have an alternative arrangement of domains, one that does not present the tight NBD1/NBD2 and NBD/TMD interfaces seen in canonical outward-facing ABC structures (71).

The remaining two highly VX-770–sensitive mutations we identify, R347P and L927P, are close to the narrowest portion of the permeation pathway, thought to constitute the CFTR gate (72, 73), and positioned adjacent to the transmembrane VX-770–binding site (18) (Figs. 5*D* and 7). Both mutations replace native side chains with prolines, which impose an unusual geometry on the peptide bond and restrict backbone flexibility (74). Arg-347, in TM6, is important for maintaining a stable conducting pathway (75, 76), whereas Leu-927 is in the unwound segment of TM8 (77, 78), underlying CFTR's unique channel function (78). The very low conductance measured after baseline activation in these mutants underscores the importance of backbone flexibility at both of these sites for normal channel opening and/or to maintain an open permeation pathway (19). We do not currently have a structural model of the fully open CFTR permeation pathway (19, 79). Homology modeling and molecular dynamics have been used to predict a possible fully open conformation (80), confirming that plasticity in TM8 and TM6 is likely involved in regular, ATP-gated channel opening. The large -fold increase in activity seen in the presence of the drug implies that VX-770 binding must allow mutant channels to bypass this requirement. Bound VX-770 might provide local molecular contacts that allow helical rearrangement and opening, via an alternative pathway or to an alternative open conformation.

Thus, our results indicate that the VX-770–bound open-channel conformation is distinct from the open conformation adopted during normal ATP-driven channel gating. A comparison with the action of G907, an antagonist of the MsbA bacterial ABC exporter (like CFTR, belonging to the Type IV ABC systems (71)) is relevant. High-resolution structures reveal that G907 binds at a transmembrane site close to the VX-770–binding site on CFTR. Despite the transmembrane-binding site, it causes an allosteric displacement of an NBD, releasing it from the conserved network of interactions generally stabilizing the NBD/TMD ball-and-socket joint (81). G907 increases anion conductance in cells expressing YFP-CFTR, consistent with its binding favoring an open-channel conformation.^1^ However, the uncoupled NBD seen in the G907-MsbA complex suggests that this open-channel conformation might not have canonical, dimerized NBDs. Hydrogen/deuterium exchange studies also suggest that NBD1 uncoupling might be associated with VX-770 binding (82). Further experiments are needed to test this hypothesis.

### Implications for pharmacological research

The main advantage of our assay consists in providing simultaneous measurements of ion-channel function and biogenesis. Being able to monitor how compounds or mutations affect both the number of channels at the membrane and conductance can allow deconvolution of effects on processing from those influencing gating and permeation of the channel. Describing each CF-causing mutation with two coordinates (ρ and *G*_CFTR_) is a more informative way of characterizing mutations (*e.g.* see Fig. 6) and how they respond to drugs (*e.g.* see Fig. 5*D*) than using either functional or surface-exposure measures alone. The higher information content of measurements will accelerate discovery in projects investigating molecular mechanisms. For instance, using mutagenesis to scan secondary structure elements or to target residues in putative drug-binding sites, hypotheses can be generated or tested rapidly, and results will pinpoint areas worthy of further investigation by more labor-intensive techniques (*e.g.* patch-clamp/molecular dynamics).

In addition to providing a valuable tool for basic science investigation, our assay could also have a translational impact. Whereas other functional assays in more native systems (*e.g.* short-circuit current measurements on primary human bronchial epithelia, forskolin-induced swelling of intestinal organoids (83)) will remain fundamental for preclinical testing of CFTR-targeting drugs, our assay can usefully complement these.

First, the assay could be useful for the development of better-precision medicines for CF treatment. Each of the CFTR variants associated with CF could idiosyncratically affect folding, trafficking, stability, gating dynamics, and/or permeation—as well as how these properties respond to modulator drugs. A number of modulators are currently approved or in the development pipeline, and therapies combining multiple correctors and potentiators appear to be most effective, at least for patients carrying the F508del mutation (16, 17, 84). However, potentiators can negatively interfere with corrector action, and drug-drug interactions are genotype-specific (30, 31, 54). Because each mutation, other than F508del, is extremely rare, preclinical studies using our assay could provide a first molecular characterization of how individual CFTR variants respond to modulator drugs and drug combinations in controlled, simplified conditions. Such data can be very valuable to inform drug development, trial design, and therapy choice, especially for genotypes found only extremely rarely in the population (85).

Second, the assay could help develop very effective dual-activity modulator drugs for CF treatment. Both gating/permeation and processing defects likely stem from impaired folding, at least for the common F508del-CFTR variant (86). However, practical implementation of distinct potentiator and corrector screens might have so far biased the drug development process by selecting compounds for improvement only in one dimension (87). Screening using our integrated assay, by maintaining the requirement for simultaneous reduction of both defects, will maximize the chances of identifying ligands capable of redressing the primary folding defect. By shifting therapy closer to the root cause of disease, such a drug would likely reduce the need for prevention/treatment of comorbidities and exacerbations, as well as decrease the likelihood of long-term safety and tolerability problems.

Finally, CFTR plays an important role controlling fluid movement across several epithelia (2, 88), and it has been implicated in a number of pathologies, including secretory diarrheas (89), chronic obstructive pulmonary disease (90, 91), polycystic kidney disease (92), and others (93, 94). It is likely that, given the complexity of CFTR folding (8, 86), many CFTR-targeting compounds will alter its cellular processing (*e.g.* see Ref. 10), suggesting that the assay could also be usefully deployed as part of the development of novel CFTR-targeting compounds for treatment of other diseases, beyond CF.

## Experimental procedures

### Construction of the pIRES2-mCherry-YFPCFTR plasmid

The pIRES2-mCherry-YFPCFTR plasmid was obtained with two sequential subcloning steps. First, a 1.727-kb region of pcDNA3.1-YFP-CFTR (24), containing the YFP-coding sequence, was subcloned into pIRES-eGFP-CFTR, a gift from David Gadsby (Rockefeller University), using the NheI and BlpI restriction sites. Subsequently, a 0.737-kb region from pIRES2-mCherry-p53 deltaN (95) (Addgene), containing the mCherry-coding segment and part of the IRES, was subcloned into the pIRES-eGFP-YFPCFTR plasmid using the NotI and BmgBI/BtrI restriction sites. This resulted in the pIRES2-mCherry-YFPCFTR plasmid, with the IRES2 positioned between the two open reading frames for YFP-CFTR and mCherry.

To generate the rare-mutation panel, point mutations were introduced in the pIRES2-mCherry-YFPCFTR plasmid using site-directed mutagenesis (QuikChange protocol, Stratagene).

### HEK293 cell culture, transfection, and incubation

HEK293 cells were maintained in Dulbecco's modified Eagle's medium, supplemented with 2 mm l-glutamine, 100 units/ml penicillin and streptomycin, and 10% fetal bovine serum (all from Life Technologies, Inc.). Cells were seeded in poly-d-lysine–coated, black-walled 96-well plates (Costar, Fisher Scientific) and transiently transfected with the pIRES2-mCherry-YFPCFTR plasmid using Lipofectamine 2000 (Life Technologies), following the manufacturer's instructions. After transfection, cell plates were returned to the 37 °C incubator for 24 h. Prior to imaging, plates were incubated for another 24 h, at 37 or 28 °C, in 100 μl of Dulbecco's modified Eagle's medium including DMSO (vehicle), 10 μm VX-809, or 10 μm VX-770 plus 10 μm VX-809 (Selleck Chemicals), as indicated. The assay is currently run using 96-well plates, but small changes could make it compatible with a 384-well plate format.

### Image acquisition

Before imaging, cells were washed twice with 100 μl of standard buffer (140 mm NaCl, 4.7 mm KCl, 1.2 mm MgCl_2_, 5 mm HEPES, 2.5 mm CaCl_2_, 1 mm glucose, pH 7.4). The ImageXpress Micro XLS (Molecular Devices), an automated inverted wide-field fluorescence microscope with a temperature-controlled chamber (set to 37 or 28 °C, as indicated), was used for image acquisition. Protocols for automated fluid additions, enabled by a robotic arm, were created using MetaXpress software (Molecular Devices). For imaging of YFP-CFTR, a 472 ± 30-nm excitation filter and a 520 ± 35-nm emission filter were used. Excitation/emission filters at 531 ± 20 and 592 ± 20 nm were used for imaging of mCherry. Before image acquisition on each channel, the system allows an adjustment of the laser intensity and exposure time to maximize the signal while not exceeding the linear range.

For localization of CFTR, a ×60 objective was used to take nine 16-bit images per well of both fluorophores. To evaluate CFTR function, a ×20 objective was used. Two 16-bit images of mCherry were taken, one at the start and one the end of the protocol. In addition, 16-bit images of the YFP fluorescence were taken at an acquisition frequency of 0.5 Hz. For the monitoring of nonstationary CFTR activity (protocol A; see below), after 20 s, 50 μl of 300 mm I^−^ buffer (300 mm NaI, 4.7 mm KCl, 1.2 mm MgCl_2_, 5 mm HEPES, 2.5 mm CaCl_2_, 1 mm glucose, pH 7.4) was added to the standard buffer, so that the final concentration of I^−^ in the extracellular medium was 100 mm. Another 40 s later, a further 50 μl of a 100 mm I^−^ buffer containing 40 μm forskolin (100 mm NaI, 4.7 mm KCl, 1.2 mm MgCl_2_, 5 mm HEPES, 2.5 mm CaCl_2_, 1 mm glucose, 40 μm forskolin, pH 7.4) was added, so that the final concentration of forskolin in the extracellular medium was 10 μm, whereas the concentration of other components remained unaltered. For the quantification of CFTR activity at steady state (protocol B; see below), after 20 s of imaging, CFTR was activated, in the absence of extracellular I^−^, by the addition of 50 μl of standard buffer containing activating compounds (forskolin or forskolin + VX-770, both to reach final concentrations of 10 μm). After a further 230 s, by which time CFTR is assumed to be gating at steady state (39), extracellular I^−^ was raised to 100 mm (final concentration) by adding 50 μl of I^−^ buffer (as standard buffer with 140 mm NaCl replaced with 400 mm NaI). Images were taken for another 40 s. Activating compounds were also included in the second addition so as not to alter final extracellular concentrations.

### Image analysis

Image analysis was automated using MATLAB mathematical computing software (MathWorks). Separate analysis protocols were implemented to estimate CFTR membrane proximity and ion-channel function.

#### CFTR membrane proximity

First, mCherry images were binarized, and basic morphological operations (opening, closing, area opening, and dilation) were carried out to reduce noise. A distance transform with locally imposed minima was used to segment images by means of a watershed transformation and define cell boundaries. Cells were removed from analysis if they had an area of <108 μm^2^ or >5400 μm^2^, if they had a major axis length of >32.4 μm, if the area over perimeter was <25 or >300, and if they were touching the edge of the image. A 1.08 μm band, 10 or 5 pixels wide (depending on the resolution of the image), within the border of each cell was defined as the membrane-proximal zone.

Background was selected by inverting the binarized and morphologically opened mCherry image, after which it was morphologically closed using a large structuring element, to prevent cells from being selected as background. Average background intensity was then subtracted from each pixel, and the YFP and mCherry fluorescence intensity of each cell was normalized to the median YFP and mCherry fluorescence intensities of cells expressing WT-CFTR on the same plate. If the average normalized fluorescence intensity fell below 0 (due to low transfection efficiency and high background noise), cells were removed from analysis.

To estimate CFTR membrane proximity for each cell (defined as ρ; see “Results”), the average normalized YFP fluorescence intensity within the membrane-proximal zone was divided by the average normalized mCherry fluorescence over the entire cell.

(Eq. 2) $$\rho =\frac{F_{YFPmembrane}}{F_{mCherrycell}}$$

#### CFTR ion-channel function

For assessment of CFTR function, two different protocols were used. For both, cells were selected based on the mCherry fluorescence images that were taken at the beginning and at the end of the protocol. The images were binarized using an adaptive threshold, after which they were dilated and combined to account for possible minor movement of cells during the time course.

##### Protocol A: Monitoring of nonstationary CFTR activity

The within-cell YFP fluorescence at the time point before the addition of I^−^ (*F*_YFPmax_) was used to normalize within-cell YFP fluorescence intensity. The concentration of I^−^ inside the cells ([I^−^]_in_) can be estimated with Equation 3 (24), in which the binding affinity for I^−^ (*K_I_*) to YFP(H148Q/I152L) is set to 1.9 mm (23) and the normalized fluorescence intensity over time (*F*(*t*) = *F*_YFP_/*F*_YFPmax_) is determined experimentally.

(Eq. 3) $${\left[I^{-}\right]}_{in}=K_{I}\frac{\left(1-F(t)\right)}{F(t)}$$

Data was collected every 2 s, so the change [I^−^]_in_ observed at each time point can be estimated and used to calculate the rate of I^−^ entry (in mm/s) as follows.

(Eq. 4) $$\frac{\Delta {\left[I^{-}\right]}_{in}}{\Delta t}=\frac{{\left[I^{-}\right]}_{in}(t)-{\left[I^{-}\right]}_{in}(t-1)}{2s}$$

The maximal observed rate of I^−^ entry is used as a measure of cellular anion conductance. To determine whether there was increased CFTR-mediated anion conductance, the maximal rate of I^−^ entry after the addition of forskolin (which activates CFTR due to increased phosphorylation by cAMP-dependent protein kinase) was compared with the maximal rate of I^−^ entry after the addition of DMSO (vehicle, negative control).

##### Protocol B: Quantification of CFTR activity at steady state

CFTR activation (by the addition of 10 μm forskolin with or without 10 μm VX-770, as indicated) was first allowed to reach steady state in the absence of I^−^, and then quenching of YFP (again expressed as *F*_YFP_/*F*_YFPmax_) in the 40 s following extracellular I^−^ addition was measured. A simple mathematical model was used to fit observed fluorescence quenching and estimate CFTR conductance as described (39). Briefly, the model includes four free parameters: CFTR conductance at steady state (*G*_CFTR_); membrane potential at steady state, immediately prior to I^−^ addition (*V*_M_); and conductance (*G*_trans_) and time constant (τ_trans_) of a transient, endogenous non-CFTR anion conductance. The values of the four parameters were estimated by minimizing the sum of squared residuals obtained by comparing the time course of the observed *F*_YFP_/*F*_YFPmax_ with the proportion of anion-free YFP chromophore predicted by the model (also normalized to the time point before I^−^ addition). However, when the quenching time course was too fast and did not provide enough information to uniquely identify all four parameters, the value of the latter two parameters (*G*_trans_ and τ_trans_) was constrained to the average values obtained with negative controls, and only *G*_CFTR_ and *V*_M_ were left free to vary (39). Experimental data are well-described by the model, suggesting that YFP chromophore molecules, whether fused to CFTR inserted in intracellular vesicles or in the plasma membrane, behave as a single population.

For both protocol A and B, the value obtained from analysis of the observed *F*_YFP_/*F*_YFPmax_ time curves (*G*_CFTR_ and (Δ[I^−^]_in_/Δ*t*), respectively) was corrected to account for variations in transfection efficiency. Thus, the metric reporting ion-channel function was normalized for each condition/genotype by dividing by the mean *F*_mCherry_ within the cell selection (which, in turn, was normalized to *F*_mCherry_ measured for WT in the same plate).

### Statistical analysis

To determine whether the observed differences in ρ, Δ[I^−^]_in_/Δ*t*, or *G*_CFTR_ resulting from experimental manipulation and/or mutations were statistically significant, we performed either independent or paired *t* tests (pairing different genotypes/conditions measured in the same multiwell plate). When required, either a Bonferroni or a Benjamini–Hochberg correction was applied to adjust for multiple comparisons. Data in graphs represent mean ± S.E.M., and the significance level was prespecified as α = 0.05. Statistical analysis was carried out using MATLAB (MathWorks), SigmaPlot (Systat Software), SPSS (IBM), or Excel (Microsoft).

## Data availability

Most data are presented in the figures of the main article. In addition, the supporting information includes a comparison between our results for the rare-mutation panel and published data (Fig. S1), information on the statistical analyses performed (Tables S1–S6), and paired *t* test plots and distributions of log_10_ρ values for each mutant in the rare-mutation panel (Fig. S2). Analysis code and example images to run it on are provided for readers. All of the necessary instructions and files can be found at: https://github.com/stellaprins/CFTRimg. All remaining data are contained within the article.

## Footnotes

3 De Jonge, H., Ardelean, M., Bijvelds, M., and Vergani, P. (2020) Strategies for CFTR inhibition: from underlying molecular mechanisms to treatments for secretory diarrhoeas. FEBS Letters.
