# Supplementary material for: Fluorescence assay for simultaneous quantification of CFTR ion-channel function and plasma membrane proximity
Source: J Biol Chem. 2021 Jan 13;295(49):16529–44. doi: 10.1074/jbc.RA120.014061 (PMC7864054; doi:10.1074/jbc.RA120.014061)
Supplement: Supplementary file 1 [file mmc1.pdf]

Fluorescence assay for simultaneous quantification of CFTR ion-channel function and plasma membrane proximity

Supporting Information

Stella Prins[1], Emily Langron[1], Cato Hastings[2], Emily J. Hill[1], Andra C. Stefan[3], Lewis D. Griffin[2] and Paola Vergani[1]*

[1] Department of Neuroscience, Physiology and Pharmacology

[2] CoMPLEX

[3] Natural Sciences

University College London

Gower Street

WC1E 6BT London UK

Supporting Figure S1

Rare mutation panel characterization – comparison between results obtained in this study and published datasets. Comparison between ion channel function vs. short-circuit Ussing Chamber measurements (**A, C, E, G**) and membrane density vs. immunoblot studies (**B, D, F, H**). (**A-D**) Measurements obtained in this study are compared to those reported in Yu et al. J Cyst Fibros. 11: 237-45 (2012) and Van Goor et al. J Cyst Fibros. 13, 29-36 (2014). (**E-H)**. Comparisons between this study vs. Sosnay et al. Nat Genet. 45, 1160-1167 (2013). Note that different sets of genotypes were included in the different studies, and individual genotypes are not shown in the same colour in Figures 4, S1 (A-D) and S1 (E-H). For display purposes, the two conductance and the ρ axes are shown with logarithmic scaling. With these transformations, squared correlation coefficients obtained by simple linear regression (r^2) are: 0.61 (A), 0.53 (B), 0.58 (E) and 0.74 (F). Using untransformed measurements, the corresponding r^2values are: 0.61 (A), 0.49 (B), 0.68 (E) and 0.66 (F).

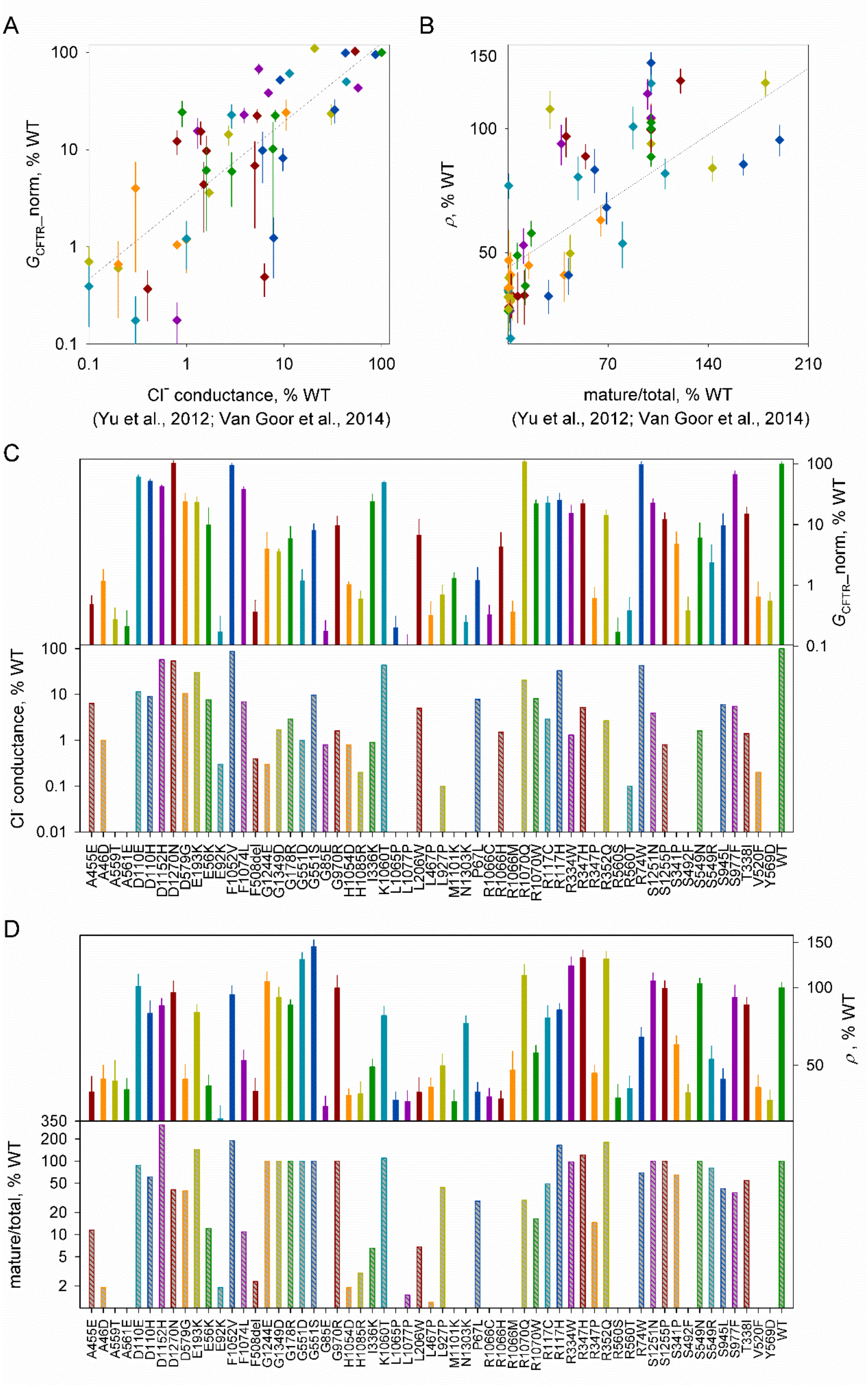

A
B
C
D
G_{CFTR}_norm, % WT
Cl⁻ conductance, % WT
(Yu et al., 2012; Van Goor et al., 2014)
ρ, % WT
mature/total, % WT
(Yu et al., 2012; Van Goor et al., 2014)
A455E A46D A559T A561E D110E D110H D1152H D1270N D579G E193K E56K E92K F1052V F1074L F508del G1244E G1349D G178R G551D G551S G85E G970R H1054D H1085R I336K K1060T L1065P L1077P L206W L467P L927P M1101K N1303K P67L R1066C R1066H R1066M R1070Q R1070W R117C R117H R334W R347H R347P R352Q R560S R560T R74W S1251N S1255P S341P S492F S549N S549R S945L S977F T338I V520F Y569D WT

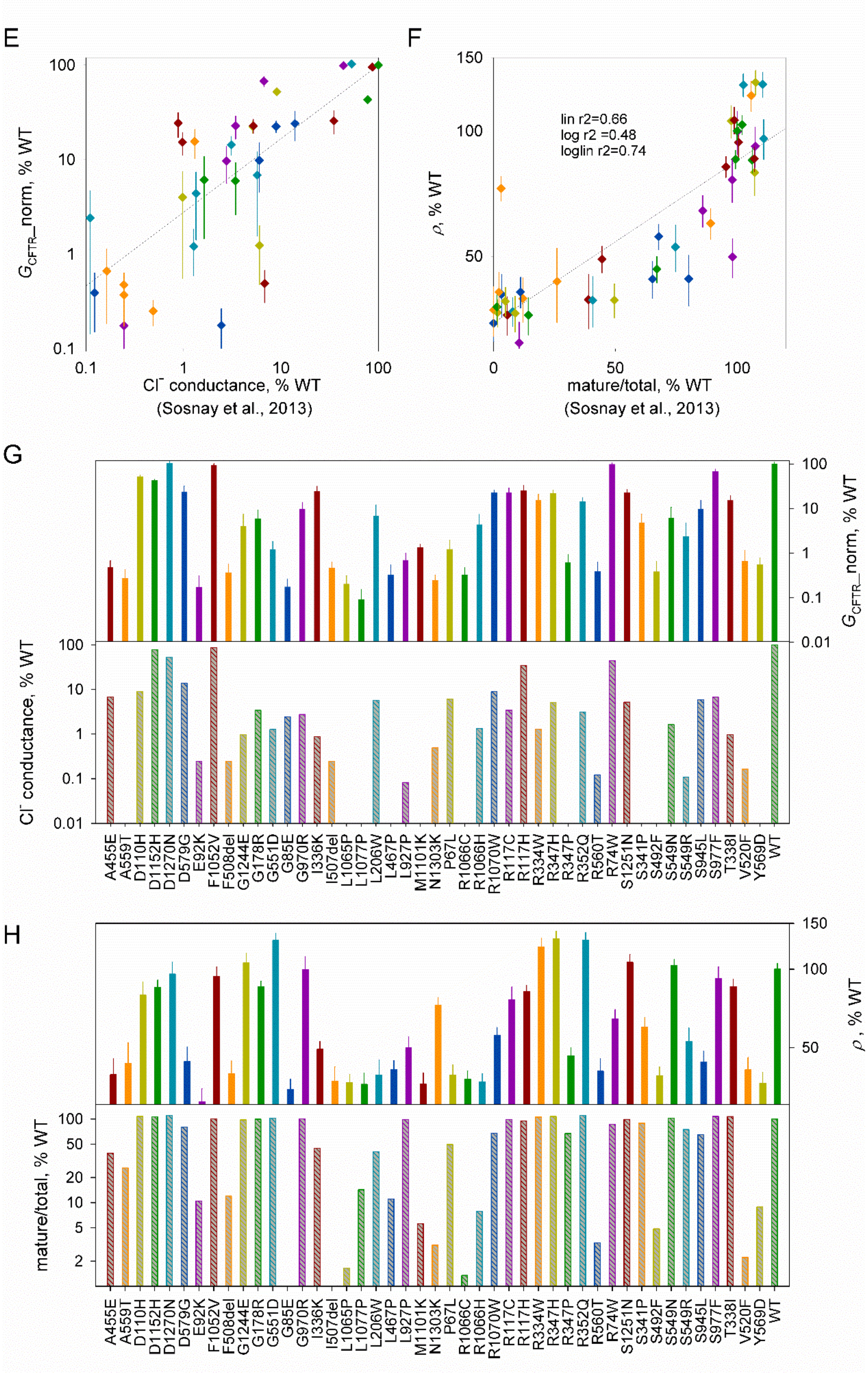
E
F
G
H
G_{CFTR}_norm, % WT
Cl⁻ conductance, % WT
(Sosnay et al., 2013)
ρ, % WT
mature/total, % WT
(Sosnay et al., 2013)
lin r2=0.66
log r2 =0.48
loglin r2=0.74
A455E A559T D110H D1152H D1270N D579G E92K F1052V F508del G1244E G178R G551D G85E G970R I336K I507del L1065P L1077P L206W L467P L927P M1101K N1303K P67L R1066C R1066H R1070W R117C R117H R334W R347H R347P R352Q R560T R74W S1251N S341P S492F S549N S549R S945L S977F T338I V520F Y569D WT

Supporting Table S1 Paired Sample t-tests comparing the $\log_{10}\rho$ of F508del-CFTR or F508del/R1070W-CFTR after different chronic incubation conditions. An independent t-test was performed to assess the significance of the difference in $\log_{10}\rho$ of WT-CFTR and F508del-CFTR at 37 °C and 28 °C. P-values were Bonferroni adjusted to account for multiple comparisons.

		Mean	SD	SD for mean difference	df	T value	Adjusted P value
37°C	F508del	-0.59	0.05	0.02	6	-4.64	0.01
	F508del + VX-809	-0.55	0.06				
	F508del	-0.58	0.07	0.02	7	-5.78	2.01E-03
	F508del/R1070W	-0.53	0.08				
	F508del + VX-809	-0.59	0.07	0.02	5	4.22	0.02
	F508del + VX-809 + VX-770 (c)	-0.63	0.06				
28°C	F508del	-0.51	0.09	0.04	6	-12.23	6.00E-05
	F508del + VX-809	-0.31	0.07				
	F508del	-0.54	0.07	0.13	6	-4.73	0.01
	F508del/R1070W	-0.40	0.10				
	F508del + VX-809	-0.34	0.08	0.20	10	4.06	4.54E-03
	F508del + VX-809 + VX-770 (c)	-0.50	0.09				
temperature correction	37°C (WT - F508del)	0.54	0.08	NA	44	5.59	4.05E-06
	28°C (WT - F508del)	0.41	0.08				

Supporting Table S2 Independent t-tests comparing the maximal rate of I^- entry after addition of 10μM forskolin vs. after DMSO (control). Cells were transfected with WT-CFTR, F508del-CFTR, or F508del/R1070W-CFTR and incubated at either 37 °C or 28 °C, with or without 10 μM VX-809, 24 hours before imaging. In some conditions the potentiator VX-770 (10 μM) was added acutely together with forskolin.

		VX-809	VX-770 (a)		Mean	SD	df	T value	P value
WT	37°C	-	-	Forskolin	0.180	0.059	16	8.65	1.99E-07
				DMSO	0.010	0.003			
		-	+	Forskolin	0.214	0.056	4	6.36	3.13E-03
				DMSO	0.008	0.003			
	28°C	-	-	Forskolin	0.137	0.023	10	13.18	1.20E-07
				DMSO	0.010	0.005			
		-	+	Forskolin	0.214	0.056	5	6.36	3.13E-03
				DMSO	0.008	0.003			
F508del	37°C	-	-	Forskolin	0.009	0.005	15	0.71	0.49
				DMSO	0.007	0.004			
		-	+	Forskolin	0.014	0.007	4	1.50	0.21
				DMSO	0.007	0.002			
		+	+	Forskolin	0.034	0.008	9	5.72	2.87E-04
				DMSO	0.013	0.004			
	28°C	-	-	Forskolin	0.012	0.009	10	0.00	1.00
				DMSO	0.012	0.014			
		-	+	Forskolin	0.059	0.042	5	2.71	0.04
				DMSO	0.004	0.001			
		+	+	Forskolin	0.124	0.039	9	6.67	9.12E-05
				DMSO	0.007	0.004			
F508del/ R1070W	37°C	-	-	Forskolin	0.044	0.026	12	2.87	0.01
				DMSO	0.014	0.010			
	28°C	-	-	Forskolin	0.090	0.032	6	4.38	4.68E-03
				DMSO	0.015	0.014			

Supporting Table S3 Independent t-tests comparing the maximal rate of I^- entry after addition of 10 µM forskolin in varying conditions. P-values were Bonferroni adjusted to account for multiple comparisons.

	Mean	SD	df	T value	P value	adjusted P value
WT 37°C	0.180	0.059	10	0.88	0.40	0.80
WT + VX-770 (a) 37°C	0.214	0.056				
WT 28°C	0.137	0.023	8	2.71	0.03	0.05
WT + VX-770 (a) 28°C	0.207	0.058				
WT 37°C	0.180	0.059	13	1.70	0.11	0.22
WT 28°C	0.137	0.023				
WT + VX-770 (a) 37°C	0.214	0.056	5	0.17	0.87	1.00
WT + VX-770 (a) 28°C	0.207	0.058				
F508del + VX-770 (a) 37°C	0.014	0.007	6	3.52	0.01	0.02
F508del + VX-770 (a) + VX809 37°C	0.034	0.008				
F508del + VX-770 (a) 28°C	0.059	0.042	7	2.34	0.05	0.10
F508del + VX-770 (a) + VX809 28°C	0.124	0.039				
F508del + VX-770 (a) + VX809 37°C	0.034	0.008	9	5.06	6.77E-04	1.35E-03
F508del + VX-770 (a) + VX809 28°C	0.124	0.039				
F508del 37°C	0.009	0.005	13	-3.70	2.69E-03	0.01
F508del/R1070W 37°C	0.044	0.026				
F508del/R1070W 37°C	0.044	0.026	9	-2.62	0.03	0.06
F508del/R1070W 28°C	0.090	0.032				

Supporting Table S4 Summary of statistical data for membrane density profiling of rare mutation panel.

Mutation	Mean ρ	SEM	n
A455E	0.327	0.027	12
A46D	0.363	0.022	12
A559T	0.371	0.047	12
A561E	0.328	0.018	13
D110E	0.827	0.044	12
D110H	0.649	0.034	11
D1152H	0.688	0.022	12
D1270N	0.782	0.043	11
D579G	0.363	0.024	12
E193K	0.65	0.021	13
E56K	0.338	0.016	11
E92K	0.254	0.014	12
F1052V	0.761	0.031	10
F1074L	0.423	0.021	11
F508del	0.315	0.02	14
G1244E	0.857	0.037	11
G1349D	0.747	0.029	12
G178R	0.69	0.017	12
G551D	1.042	0.034	12
G551S	1.167	0.033	12
G85E	0.281	0.012	12
G970R	0.815	0.045	11
H1054D	0.308	0.01	10
H1085R	0.307	0.017	9
I336K	0.398	0.015	10
I507del	0.305	0.021	11
K1060T	0.632	0.025	11
L1065P	0.296	0.01	12
L1077P	0.294	0.014	12
L206W	0.309	0.024	12
L467P	0.333	0.014	13

Mutation	Mean ρ	SEM	n
L927P	0.406	0.022	11
M1101K	0.294	0.015	11
N1303K	0.588	0.02	11
P67L	0.319	0.015	11
R1066C	0.306	0.012	11
R1066H	0.29	0.013	11
R1066M	0.402	0.038	12
R1070Q	0.899	0.041	12
R1070W	0.438	0.019	10
R117C	0.626	0.035	12
R117H	0.662	0.018	12
R1283M	0.543	0.027	11
R334W	0.985	0.041	12
R347H	1.058	0.035	12
R347P	0.377	0.013	13
R352Q	1.046	0.033	13
R560S	0.307	0.02	12
R560T	0.332	0.019	13
R74W	0.522	0.022	12
S1251N	0.859	0.033	11
S1255P	0.804	0.029	12
S341P	0.487	0.021	11
S492F	0.316	0.013	12
S549N	0.835	0.021	11
S549R	0.432	0.029	11
S945L	0.358	0.018	10
S977F	0.749	0.038	11
T338I	0.681	0.024	13
V520F	0.336	0.02	12
Y569D	0.298	0.015	13
WT	0.809	0.019	23

Supporting Figure S2

Probability density distributions for $\log_{10}\rho$ values for each CFTR mutant in the panel (orange), compared to WT-CFTR (blue). Plots on left illustrate measurements obtained for WT and mutant from individual plates, paired for statistical analysis. Asterisks indicate a significant difference ($P < 0.05$) in mean $\log_{10}\rho$ between WT and mutant, following paired t-tests and correction for multiple comparisons using Benjamini-Hochberg procedure.

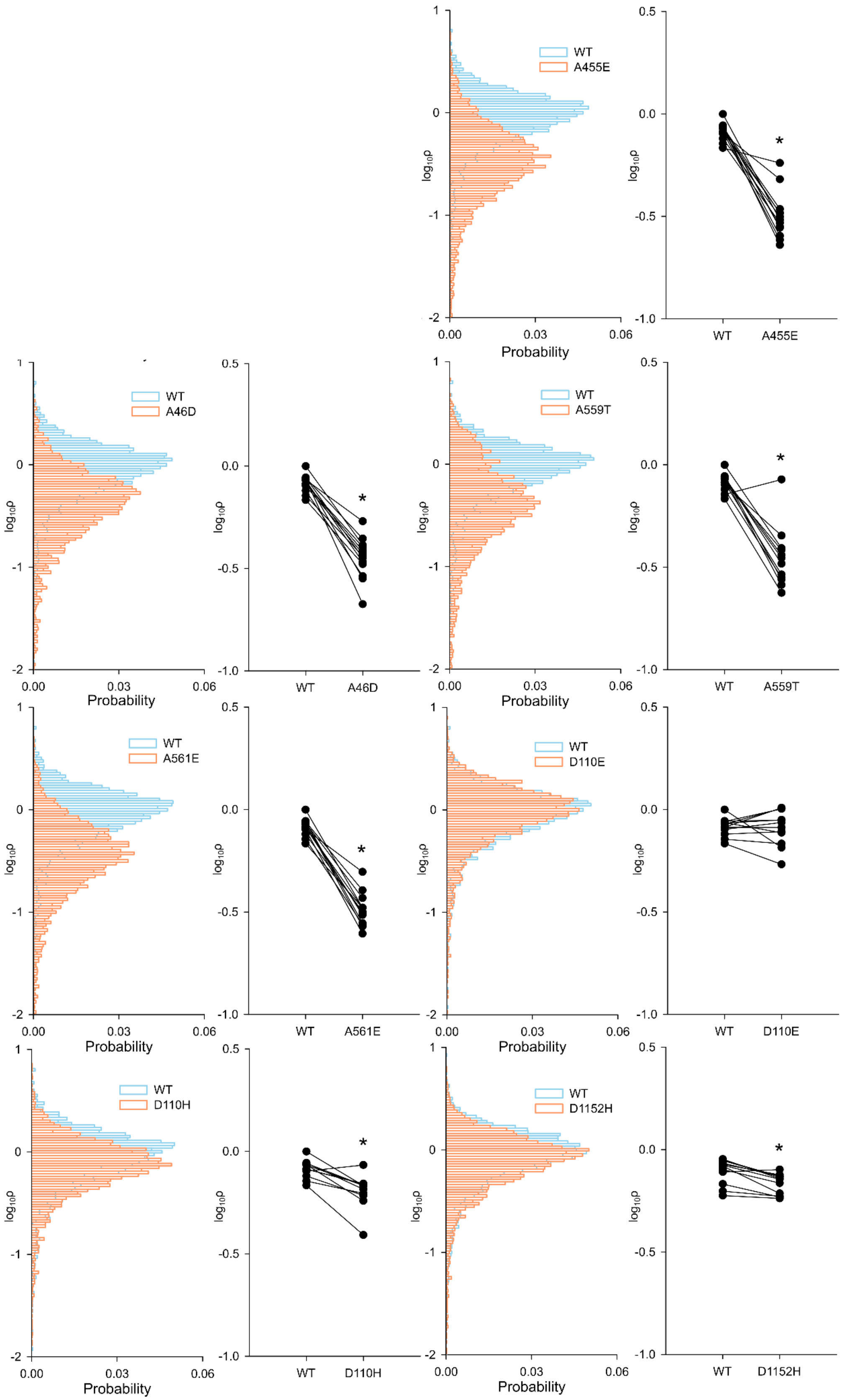
WT
A455E
WT
A46D
WT
A559T
WT
A561E
WT
D110E
WT
D110H
WT
D1152H
log10ρ
Probability
0.00
0.03
0.06
*

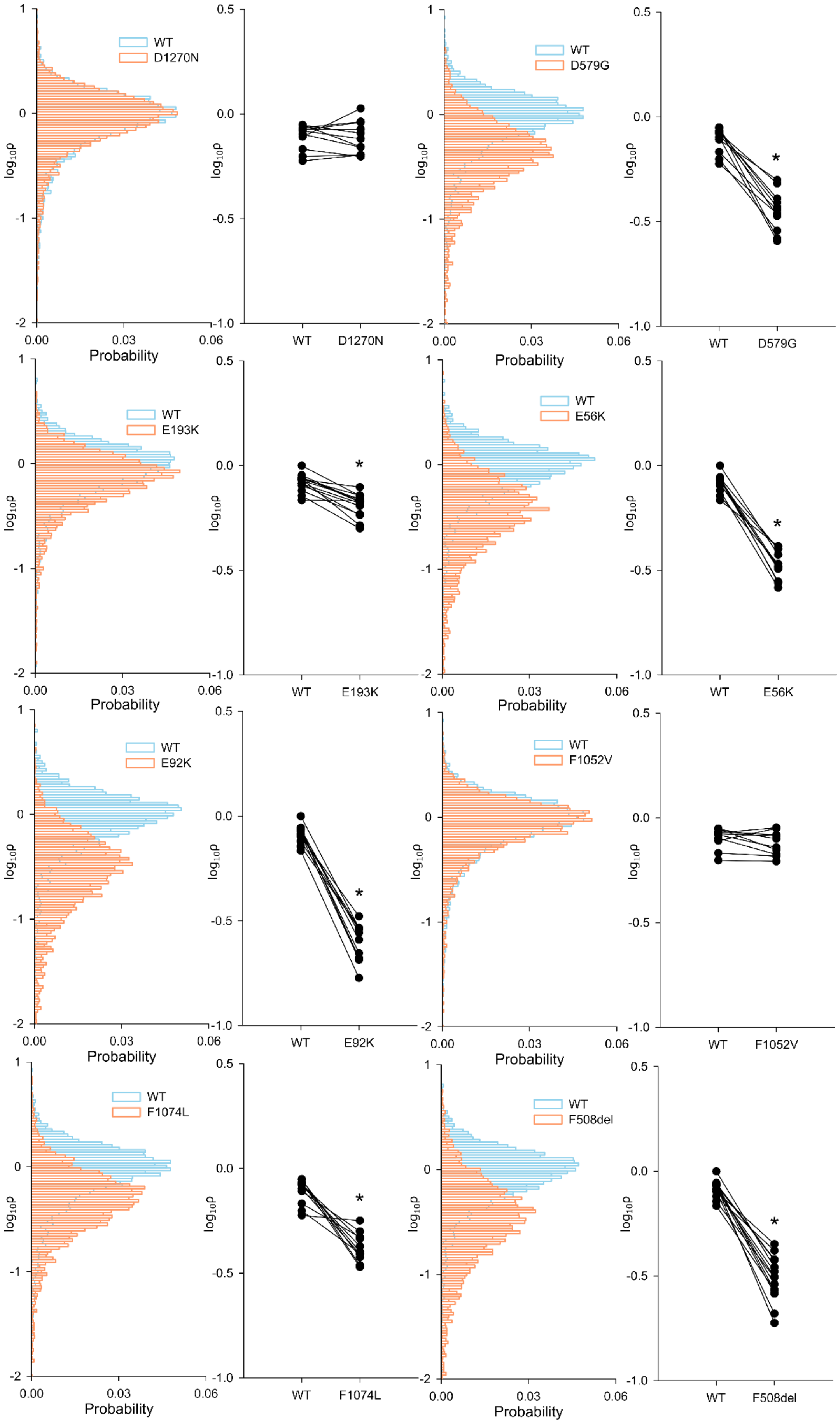
WT
D1270N
log10ρ
Probability
WT
D579G
WT
E193K
WT
E56K
WT
E92K
WT
F1052V
WT
F1074L
WT
F508del
*

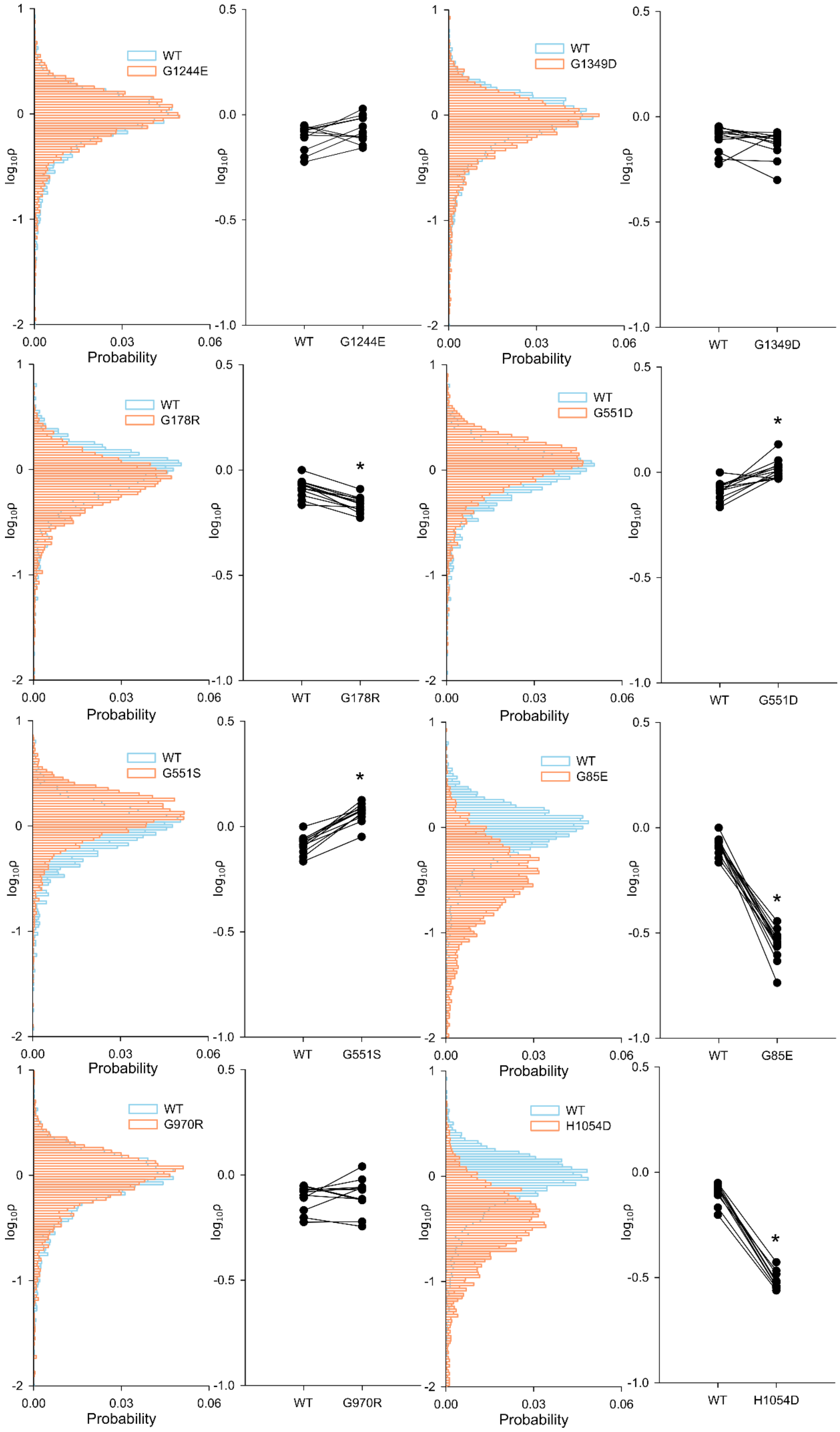
WT
G1244E
G1349D
G178R
G551D
G551S
G85E
G970R
H1054D
Probability
log10ρ
*

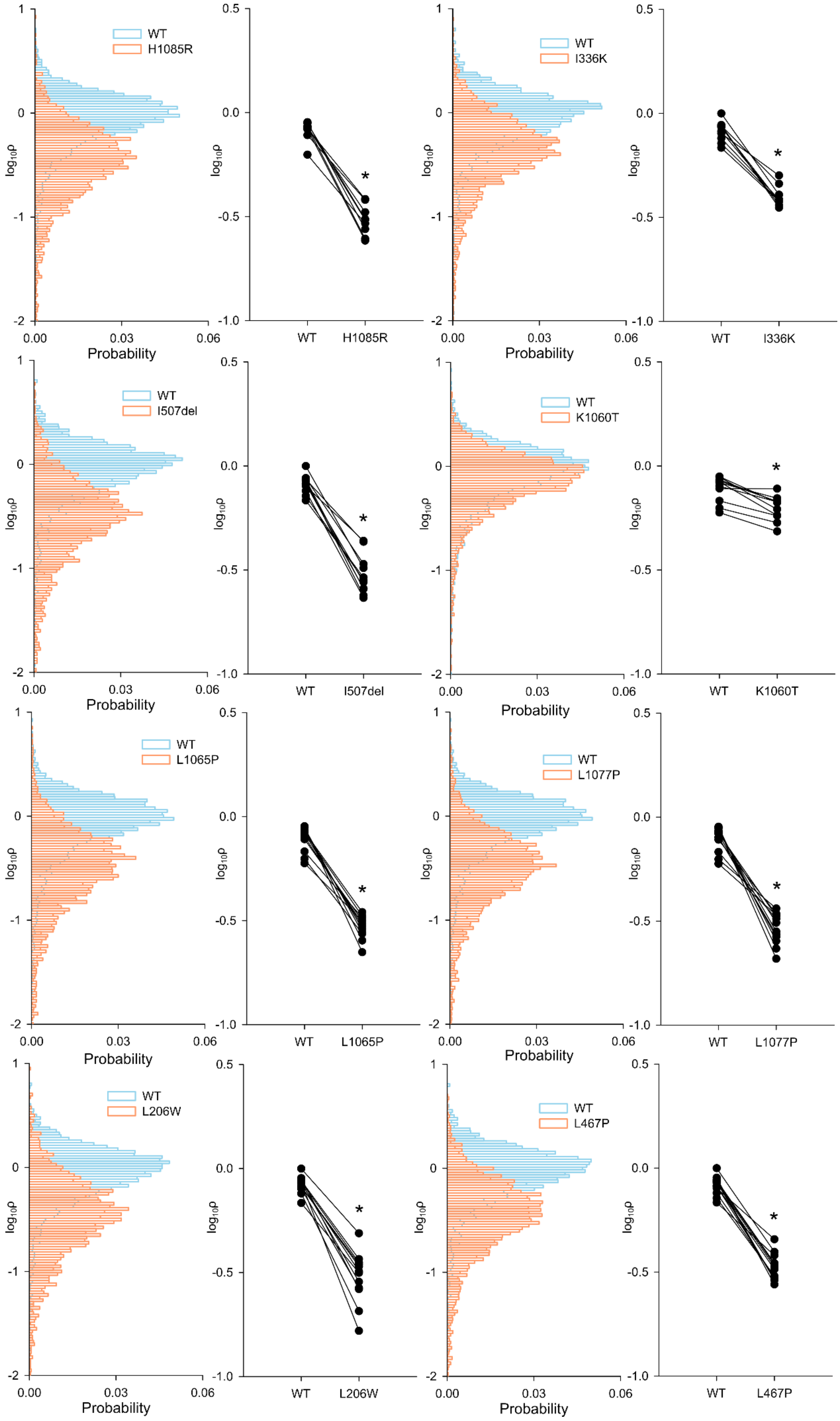
WT
H1085R
I336K
I507del
K1060T
L1065P
L1077P
L206W
L467P
Probability
$log_{10}ρ$
*
0.00
0.03
0.06
1
0
-1
-2
0.5
0.0
-0.5
-1.0

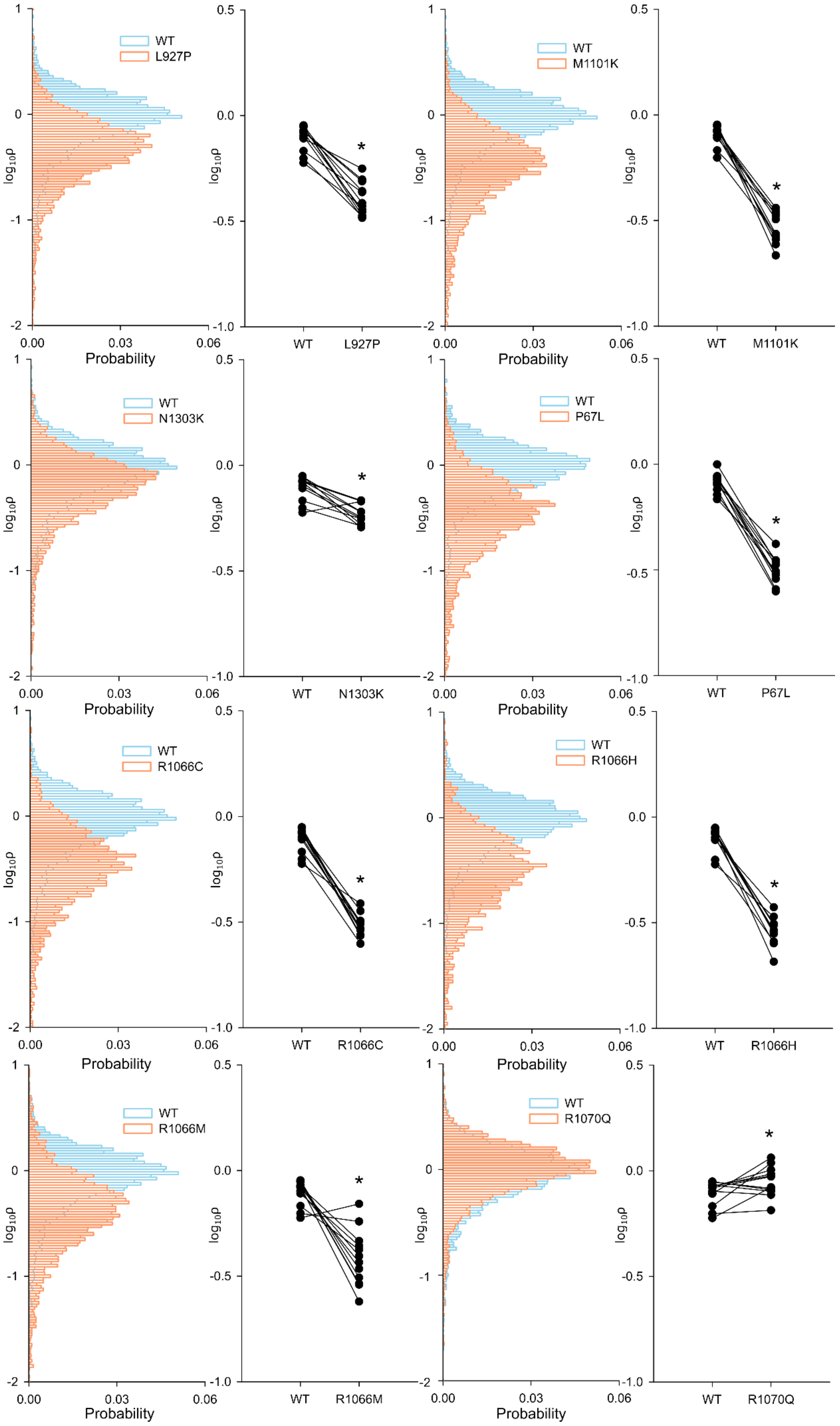
WT
L927P
WT
M1101K
WT
N1303K
WT
P67L
WT
R1066C
WT
R1066H
WT
R1066M
WT
R1070Q
Probability
log10ρ
0.00
0.03
0.06
1
0
-1
-2
0.5
0.0
-0.5
-1.0
*

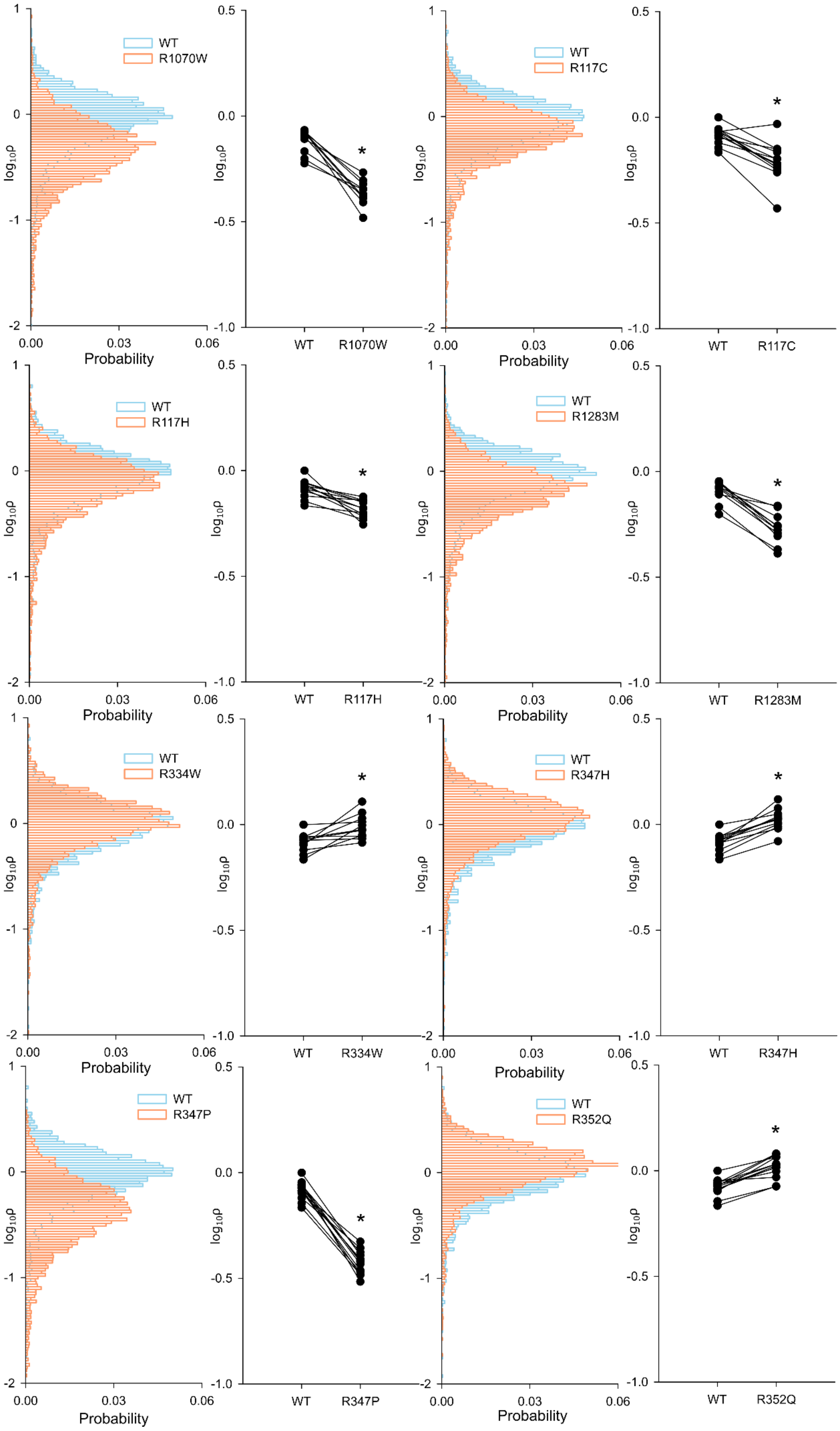
WT
R1070W
R117C
R117H
R1283M
R334W
R347H
R347P
R352Q
Probability
log10ρ
*

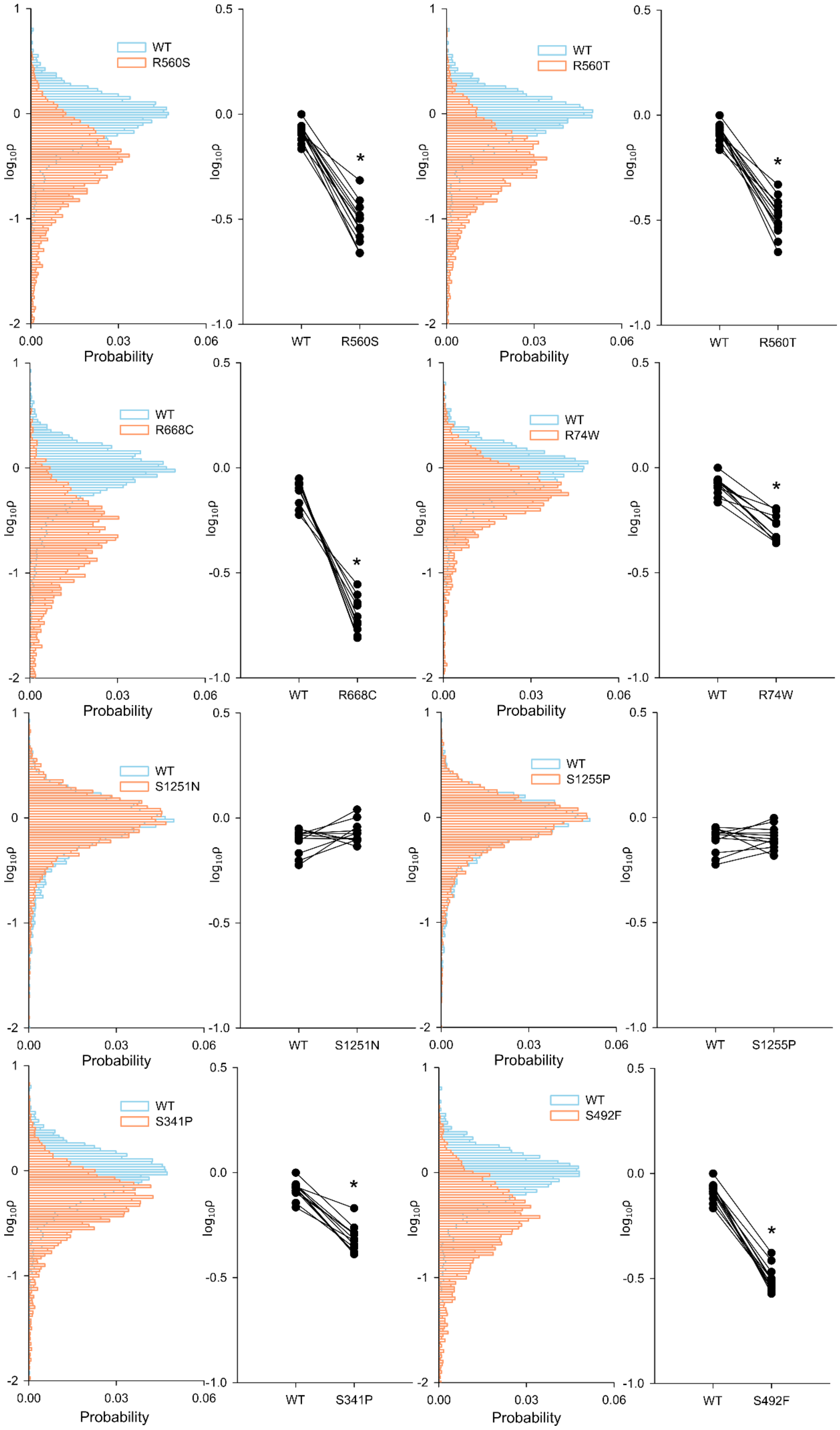
WT
R560S
WT
R560T
WT
R668C
WT
R74W
WT
S1251N
WT
S1255P
WT
S341P
WT
S492F
log10ρ
Probability
*

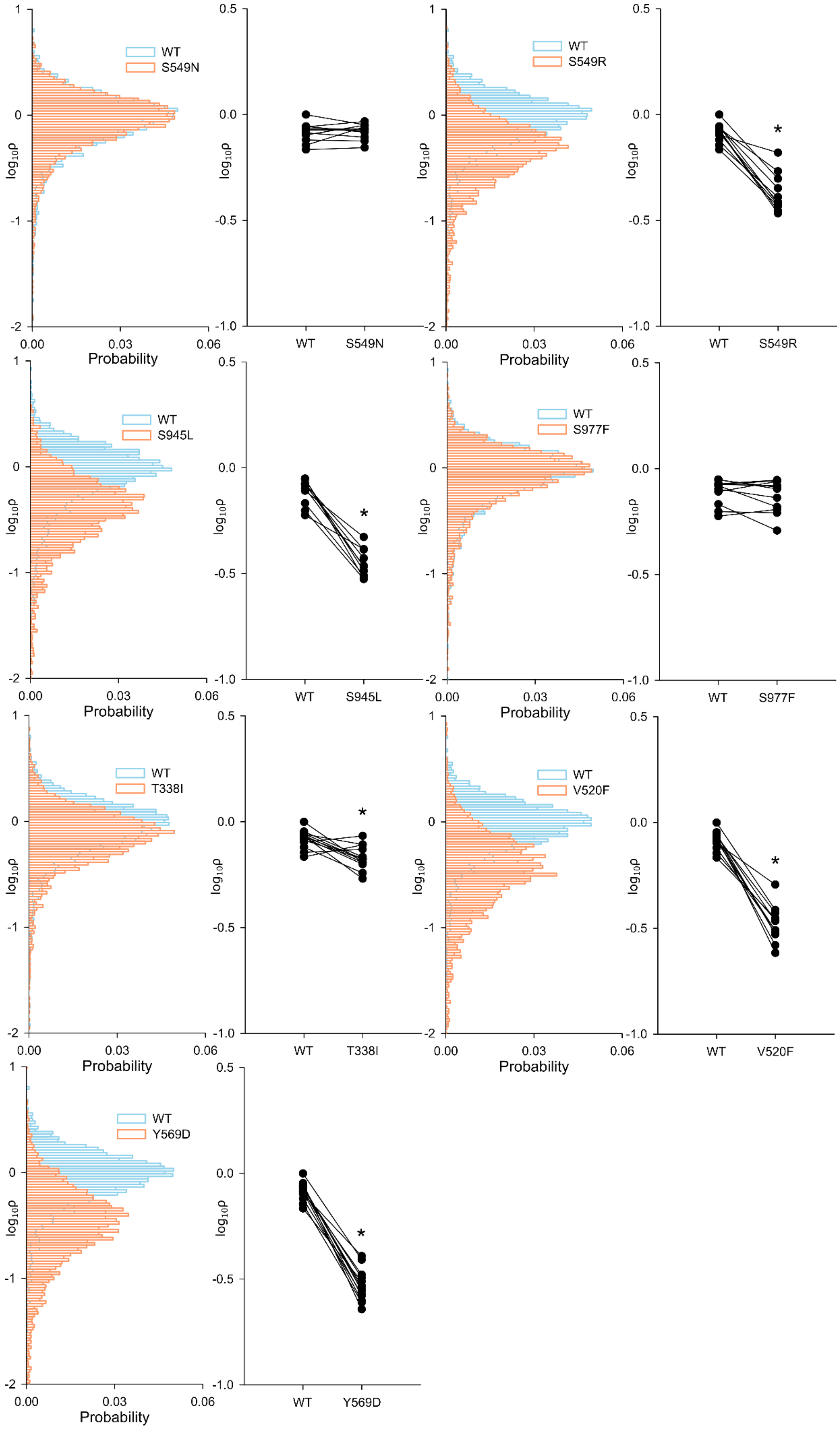
WT
S549N
log10ρ
Probability
WT
S549R
*
WT
S945L
WT
S977F
WT
T338I
WT
V520F
WT
Y569D
0.00
0.03
0.06
1
0
-1
-2
0.5
0.0
-0.5
-1.0

Supporting Table S5

Data for CFTR conductance (G_{CFTR}_norm, nS) profiling of rare mutation panel, in vehicle control conditions with no CFTR activation (**DMSO**), and after baseline activation with 10 μM forskolin (**fsk**). For each genotype, normality (Shapiro-Wilk) and equal variance preliminary tests were performed. Statistical significance of difference between control and baseline CFTR activity was assessed with a one-tailed t-test, assuming equal or unequal (marked with *) variance, depending on result of preliminary test. For R1066H and R1070W, the normality test of input groups failed and significance of within genotype difference between groups was quantified with a non-parametric Mann-Whitney Rank Sum Test.

Mutation	DMSO	SEM	n	fsk	SEM	n	P value (t test)	P value (Rank Sum test)
**A455E**	0.14	0.10	3	0.88	0.33	3	0.0490	
**A46D**	0.36	0.19	3	2.14	1.16	3	0.1035	
**A559T**	0.22	0.11	3	0.50	0.27	3	0.1890	
**A561E**	0.14	0.07	3	0.39	0.30	3	0.2340	
**D110E**	0.93	0.58	3	109.63	8.65	3	0.0001	
**D110H**	0.42	0.34	3	93.95	6.55	3	0.0001	
**D1152H**	1.13	0.49	3	77.59	3.07	3	8.14E-06	
**D1270N**	1.55	0.32	3	185.46	19.12	3	0.0003	
**D579G**	0.09	0.09	3	43.30	14.86	3	0.0220	
**E193K**	0.62	0.26	3	42.22	9.76	3	0.0254*	
**E56K**	0.25	0.25	3	18.26	16.01	3	0.1620	
**E92K**	0.14	0.14	3	0.32	0.24	3	0.2880	
**F1052V**	1.82	0.63	3	171.92	10.39	3	4.11E-05	
**F1074L**	0.98	0.44	3	69.19	6.21	3	0.0041*	
**F508del**	0.13	0.13	3	0.80	0.46	3	0.1150	
**G1244E**	0.43	0.33	3	7.20	6.21	3	0.1690	
**G1349D**	0.09	0.09	3	6.49	0.70	3	0.0004	
**G178R**	0.81	0.41	3	10.73	6.06	3	0.0890	
**G551D**	0.44	0.26	3	2.18	1.12	3	0.1349*	
**G551S**	0.42	0.21	3	14.73	3.82	3	0.0100	
**G85E**	0.63	0.63	3	0.32	0.16	3	0.3270	
**G970R**	0.44	0.24	3	17.45	7.30	3	0.0405	
**H1054D**	0.26	0.26	3	1.89	0.17	3	0.0030	
**H1085R**	0.26	0.24	3	1.08	0.37	3	0.0685	
**I336K**	0.95	0.22	3	43.76	12.94	3	0.0150	
**I507del**	0.20	0.20	3	0.85	0.29	3	0.0680	
**K1060T**	2.24	1.44	3	89.88	4.34	3	2.18E-05	

**L1065P**	0.15	0.15	3	0.37	0.19	3	0.2100	
**L1077P**	0.30	0.30	3	0.17	0.11	3	0.3475	
**L206W**	0.48	0.22	4	12.35	9.55	3	0.0990	
**L467P**	0.04	0.04	3	0.58	0.39	3	0.1200	
**L927P**	0.04	0.04	3	1.26	0.56	3	0.0799*	
**M1101K**	0.12	0.06	3	2.41	0.46	3	0.0040	
**N1303K**	0.16	0.12	3	0.45	0.13	3	0.0905	
**P67L**	0.13	0.12	3	2.23	1.37	3	0.1010	
**R1066C**	0.03	0.02	3	0.60	0.25	3	0.0435	
**R1066H**	0.85	0.37	3	7.92	5.38	4		0.057
**R1066M**	0.05	0.05	3	0.67	0.32	3	0.0650	
**R1070Q**	3.39	0.76	4	197.94	9.85	4	0.0001*	
**R1070W**	0.54	0.30	4	40.38	5.56	4		0.029
**R117C**	0.27	0.13	3	40.97	11.27	3	0.0115	
**R117H**	0.75	0.36	3	46.27	12.73	3	0.0351*	
**R1283M**	0.35	0.09	3	19.96	5.88	3	0.0145	
**R334W**	0.82	0.69	3	28.00	9.49	3	0.0230	
**R347H**	0.31	0.20	3	40.22	6.14	3	0.0015	
**R347P**	0.18	0.10	3	1.11	0.57	3	0.0915	
**R352Q**	0.30	0.12	3	25.83	5.88	3	0.0060	
**R560S**	0.04	0.04	3	0.31	0.21	3	0.1395	
**R560T**	0.24	0.21	3	0.70	0.43	3	0.1950	
**R74W**	1.13	0.39	3	178.21	16.56	3	0.0002	
**S1251N**	0.65	0.20	3	41.08	7.06	3	0.0025	
**S1255P**	0.21	0.19	3	22.07	6.06	3	0.0115	
**S341P**	0.28	0.28	3	8.80	4.85	3	0.0770	
**S492F**	0.30	0.23	3	0.69	0.48	3	0.2465	
**S549N**	0.25	0.12	3	11.00	8.37	3	0.1340	
**S549R**	0.25	0.25	3	4.35	4.09	3	0.1870	
**S945L**	0.00	0.00	3	17.70	9.55	3	0.1024*	
**S977F**	0.45	0.10	3	121.72	15.37	3	0.0005	
**T338I**	0.25	0.15	4	27.53	7.35	4	0.0050	
**V520F**	0.06	0.06	3	1.19	0.86	3	0.1290	
**Y569D**	0.10	0.09	3	1.00	0.40	3	0.0460	
**WT**	**2.53**	**0.54**	**7**	**180.31**	**15.97**	**6**	**0.0001***	

Supporting Table S6

Comparison of CFTR conductance (G_{CFTR}_norm, nS) after baseline activation with 10 µM forskolin (**fsk**) vs. potentiated activation with 10 µM forskolin + 10 µM VX-770 (a) (**fsk + VX-770**). Statistical significance of difference was assessed as described for table S6. In this dataset, normality test failed for G85E, R1066C, R1066H and S549R.

Mutation	fsk	SEM	n	fsk+ VX-770	SEM	n	P value (t test)	P value (Rank Sum test)
**A455E**	0.88	0.33	3	5.46	0.59	3	0.0005	
**A46D**	2.14	1.16	3	19.02	1.34	3	0.0002	
**A559T**	0.50	0.27	3	0.74	0.15	3	0.1215	
**A561E**	0.39	0.30	3	0.31	0.11	4	0.2018	
**D110E**	109.63	8.65	3	214.38	3.61	3	0.0001	
**D110H**	93.95	6.55	3	205.40	6.63	3	0.0001	
**D1152H**	77.59	3.07	3	213.55	13.93	3	0.0002	
**D1270N**	185.46	19.12	3	207.96	13.18	3	0.0970	
**D579G**	43.30	14.86	3	140.17	25.68	3	0.0078	
**E193K**	42.22	9.76	3	243.15	15.27	3	0.0001*	
**E56K**	18.26	16.01	3	38.96	8.62	3	0.0795	
**E92K**	0.32	0.24	3	0.79	0.28	3	0.0675	
**F1052V**	171.92	10.39	3	213.18	4.42	3	0.0055	
**F1074L**	69.19	6.21	3	152.94	5.98	3	0.0001	
**F508del**	0.80	0.46	3	4.29	0.80	3	0.0035	
**G1244E**	7.20	6.21	3	50.71	15.47	3	0.0148	
**G1349D**	6.49	0.70	3	202.73	5.63	3	1.03E-06	
**G178R**	10.73	6.06	3	232.07	25.17	3	0.0003	
**G551D**	2.18	1.12	3	35.13	7.56	3	0.0033	
**G551S**	14.73	3.82	3	207.71	35.87	3	0.0015	
**G85E**	0.32	0.16	3	0.31	0.16	3		1
**G970R**	17.45	7.30	3	183.91	7.78	3	1.63E-05	
**H1054D**	1.89	0.17	3	40.92	6.29	3	0.0008	
**H1085R**	1.08	0.37	3	28.72	5.22	3	0.0015	
**I336K**	43.76	12.94	3	139.96	20.78	3	0.0043	
**I507del**	0.85	0.29	3	0.36	0.36	3	0.0858	
**K1060T**	89.88	4.34	3	197.15	7.63	3	3.55E-05	
**L1065P**	0.37	0.19	3	2.07	0.79	3	0.0260	
**L1077P**	0.17	0.11	3	0.27	0.21	3	0.1715	

**L206W**	12.35	9.55	3	42.40	5.37	4	0.0080	
**L467P**	0.58	0.39	3	0.23	0.05	3	0.1055	
**L927P**	1.26	0.56	3	101.26	7.07	3	3.66E-05	
**M1101K**	2.41	0.46	3	8.26	3.20	3	0.0539*	
**N1303K**	0.45	0.13	3	14.93	2.59	3	0.0076*	
**P67L**	2.23	1.37	3	20.65	5.93	3	0.0098	
**R1066C**	0.60	0.25	3	1.21	0.30	3		0.4
**R1066H**	7.92	5.38	4	33.71	5.59	3		0.057
**R1066M**	0.67	0.32	3	1.31	0.21	3	0.0415	
**R1070Q**	197.94	9.85	4	214.84	2.44	3	0.0533	
**R1070W**	40.38	5.56	4	160.94	14.03	3	0.0001	
**R117C**	40.97	11.27	3	202.45	7.36	3	0.0001	
**R117H**	46.27	12.73	3	203.39	4.16	3	0.0001	
**R1283M**	19.96	5.88	3	86.07	10.10	3	0.0013	
**R334W**	28.00	9.49	3	157.23	13.48	3	0.0003	
**R347H**	40.22	6.14	3	219.57	7.75	3	1.08E-05	
**R347P**	1.11	0.57	3	23.03	8.52	3	0.0155	
**R352Q**	25.83	5.88	3	222.40	5.71	3	3.28E-06	
**R560S**	0.31	0.21	3	0.45	0.05	3	0.1400	
**R560T**	0.70	0.43	3	0.01	0.01	3	0.0627*	
**R74W**	178.21	16.56	3	193.08	1.97	3	0.1058	
**S1251N**	41.08	7.06	3	164.18	38.96	3	0.0090	
**S1255P**	22.07	6.06	3	221.11	9.61	3	1.37E-05	
**S341P**	8.80	4.85	3	42.08	13.12	3	0.0190	
**S492F**	0.69	0.48	3	9.38	7.39	3	0.0765	
**S549N**	11.00	8.37	3	193.79	10.57	3	3.51E-05	
**S549R**	4.35	4.09	3	51.69	4.75	3		0.1
**S945L**	17.70	9.55	3	125.69	8.66	3	0.0003	
**S977F**	121.72	15.37	3	204.79	10.83	3	0.0030	
**T338I**	27.53	7.35	4	84.76	6.72	3	0.0008	
**V520F**	1.19	0.86	3	0.48	0.29	3	0.1190	
**Y569D**	1.00	0.40	3	0.20	0.11	3	0.0310	
**WT**	**180.31**	**15.97**	**6**	**218.00**	**33.27**	**4**	**0.0715**	

Supporting Table S7

Estimated values for fit parameters in traces for Figure 4B.

**Mutation**	**condition**	G_{CFTR} (nS)	V_M (mV)	G_{trans} (nS)	τ_{trans} (s)
**WT**	fsk	152.88	-59.42	14.8	5
**S492F**	DMSO	0.73	-79.89	14.8	5
	fsk	1.603	-69.19	14.8	5
	fsk+VX-770	24.07	-85.18	14.8	5
**H1085R**	DMSO	0.35	-42.37	14.8	5
	fsk	1.28	-51.01	19.1	3.94
	fsk+VX-770	35.02	-76.3	14.8	5
**H1054D**	DMSO	0.000125	-58.76	14.8	5
	fsk	2.29	-50.85	14.8	5
	fsk+VX-770	30.17	-70.69	14.8	5
**L927P**	DMSO	0.0000259	-85.1	14.8	5
	fsk	1.47	-41.2	14.8	5
	fsk+VX-770	103.2	-60.49	14.8	5
**R1283M**	DMSO	0.57	-75.0	17.4	4
	fsk	25.86	-75.3	14.8	5
	fsk+VX-770	70.65	-50.4	14.8	5

Supporting Table S8

Distances measured between α-carbons of highly VX-770 sensitive mutation sites and charged residues in the vicinity as measured in the PDB ID 6MSM structure.

**Mutation**	location	interacting charge	location	distance(Å)
**G178R**	TM3, ICL1	K254<br>E257<br>R258	TM4, ICL2	9.876<br>6.657<br>7.913
**H1045D**	ICL4	E543	NBD1, X-loop	8.875
**H1085R**	TM11	R1048	TM10	6.277
**N1303K**	NBD2	R1358	NBD2	7.843
**G1349D**	NBD2	γ-phosphate	ATP, site 1	6.173
